# Engineering a niche supporting hematopoietic stem cell development using integrated single-cell transcriptomics

**DOI:** 10.1038/s41467-022-28781-z

**Published:** 2022-03-24

**Authors:** Brandon Hadland, Barbara Varnum-Finney, Stacey Dozono, Tessa Dignum, Cynthia Nourigat-McKay, Adam M. Heck, Takashi Ishida, Dana L. Jackson, Tomer Itkin, Jason M. Butler, Shahin Rafii, Cole Trapnell, Irwin D. Bernstein

**Affiliations:** 1 Clinical Research Division, Fred Hutchinson Cancer Research Center, Seattle, WA 98109 USA; 2 Department of Pediatrics, University of Washington School of Medicine, Seattle, WA 98105 USA; 3 Department of Genome Sciences, University of Washington School of Medicine, Seattle, WA 98105 USA; 4 Department of Genetic Medicine, Ansary Stem Cell Institute, Howard Hughes Medical Institute, Weill Cornell Medical College, New York, NY 10021 USA; 5 Center for Discovery and Innovation, Hackensack University Medical Center, Nutley, NJ 07110 USA

**Keywords:** Haematopoiesis, Haematopoietic stem cells

## Abstract

Hematopoietic stem cells (HSCs) develop from hemogenic endothelium within embryonic arterial vessels such as the aorta of the aorta-gonad-mesonephros region (AGM). To identify the signals responsible for HSC formation, here we use single cell RNA-sequencing to simultaneously analyze the transcriptional profiles of AGM-derived cells transitioning from hemogenic endothelium to HSCs, and AGM-derived endothelial cells which provide signals sufficient to support HSC maturation and self-renewal. Pseudotemporal ordering reveals dynamics of gene expression during the hemogenic endothelium to HSC transition, identifying surface receptors specifically expressed on developing HSCs. Transcriptional profiling of niche endothelial cells identifies corresponding ligands, including those signaling to Notch receptors, VLA-4 integrin, and CXCR4, which, when integrated in an engineered platform, are sufficient to support the generation of engrafting HSCs. These studies provide a transcriptional map of the signaling interactions necessary for the development of HSCs and advance the goal of engineering HSCs for therapeutic applications.

## Abstract (web summary)

Here, the authors use single cell RNA-sequencing to generate an atlas of signaling interactions regulating embryonic hematopoietic stem cell (HSC) development and apply this knowledge to engineer a niche sufficient to support HSC maturation in vitro.

## Introduction

One of the longstanding challenges in the field of hematopoiesis has been identifying the unique signal pathways and transcriptional programs that orchestrate the formation of long-term engrafting hematopoietic stem cells (HSCs). Complicating the study of HSC formation, embryonic hematopoiesis proceeds through sequential waves of lineage-restricted primitive, erythroid/myeloid, and lymphoid/myeloid progenitors prior to the emergence of the first HSCs^1^. During hematopoietic development, specialized endothelial-like precursor cells, termed hemogenic endothelium (HE), simultaneously downregulate endothelial-specific and activate hematopoietic-specific transcriptional programs to give rise to hematopoietic progenitors and HSCs. For functional long-term engrafting HSCs to emerge, HE must acquire and maintain HSC-defining properties such as the ability to self-renew, home, and provide multilineage hematopoiesis, properties which distinguish rare HSCs from other embryonic hematopoietic progenitors. Currently, the combination of cell-intrinsic and extrinsic signals necessary and sufficient to support the acquisition of these HSC-defining properties during embryonic development from HE remains inadequately defined, knowledge of which is essential for the goal of engineering HSC development ex vivo.

The emergence of HSCs occurs uniquely in the context of arterial vessels such as the aorta of the embryonic region referred to as the aorta-gonad-mesonephros (AGM)^2–4^. The arterial environment in which HSCs develop suggests cell-intrinsic properties of “arterialized” HE and cell-extrinsic signals from the arterial vascular niche may contribute to HSC fate. Supporting this concept, a number of studies have demonstrated the importance of arterial programs, such as those regulated by the Notch pathway, explicitly in definitive multilineage hematopoiesis and HSC formation^5–12^. This argues the need for further efforts to identify the precise signaling interactions between arterialized HE and the arterial vascular niche required for HSC formation.

Further complicating the study of HSCs is their scarcity relative to other progenitors arising simultaneously in the developing embryo. HSC activity, as measured by direct transplantation into adult immune-competent recipients, is first consistently detected at around embryonic day 11 (E11) in the AGM at very low frequency (~1 HSC per AGM based on limit dilution transplantation)^13^. Hemogenic precursors capable of maturation to HSC by ex vivo stromal culture or neonatal transplantation have been detected as early as E9 and increase in numbers between E10 and E11 in the AGM, prior to migration to the fetal liver where further maturation and expansion of HSCs occurs^13–15^. These HSC precursors have been characterized by their sequential expression of initial hematopoietic surface markers, CD41 followed by CD45, within the larger population of cells expressing the endothelial marker VE-Cadherin, defining a series of VE-Cadherin^+^CD41^+^CD45^−^ pro-HSC/pre-HSC I and VE-Cadherin^+^CD45^+^ pre-HSC II that mature to functional, long-term engrafting VE-Cadherin^+/−^CD45^+^ HSCs^16–18^. Although this phenotypic characterization of HSC precursors has been helpful in defining the sequence of HSC emergence, our understanding of the unique molecular properties of developing HSCs is limited by the fact that HSCs arise asynchronously from HE and remain far outnumbered by other hematopoietic progenitors with overlapping phenotypes differentiating simultaneously in intra-aortic hematopoietic clusters between E9 and E11^19^. This argues the need to study the unique properties of rare HSC precursors using single-cell techniques.

Toward this goal, advances in single-cell RNA-sequencing (scRNA-seq) technology and complementary improvements in computational algorithms to explore large, complex single-cell data sets have enabled transcriptome-wide analysis of rare stem cell populations and transitional states in development at unprecedented resolution^20^. Recent efforts have elegantly applied scRNA-seq to study the emergence of HSCs, providing new insights into the transcriptional properties of HSC precursors and supporting the notion that HSC arises from HE with arterial endothelial properties^21–26^. Building on these studies, we report here the application of single-cell transcriptomic approaches to specifically address the complex interactions between the arterial vascular niche and HE in regulating the specification and self-renewal of HSCs. We leveraged our previously reported model utilizing AGM-derived endothelial cell stroma (AGM-EC), which provides an instructive niche for the generation of engrafting HSCs from clonal embryonic precursors as early as E9^27–29^, to identify a cell surface phenotype (VE-Cadherin^+^CD61^+^EPCR^+^) enriching for HSC precursors across their developmental spectrum from HE. We then applied scRNA-seq to this enriched population, combined with in silico analysis and comparison to other single-cell transcriptomic data sets of purified HE and pre-HSC/HSC^22,26,30,31^, to study the transcriptional changes associated with HSC formation from HE in the AGM niche at single-cell resolution, with a specific focus on genes encoding cell surface receptors and downstream signaling molecules. Complementary transcriptomic analysis of the AGM-EC niche identified cognate ligands interacting with receptors on developing HSCs, which enabled us to engineer a stromal cell-independent niche that supported the generation of engrafting HSCs from embryonic hemogenic precursors in vitro. Altogether, these studies provide crucial insight into the precise conditions necessary to recapitulate the development of HSCs from HE, advancing translational efforts for de novo HSC generation.

## Results

### Single-cell RNA-sequencing identifies the transcriptional signatures of AGM-derived niche endothelial cells supporting HSC emergence

**Fig. 1:**
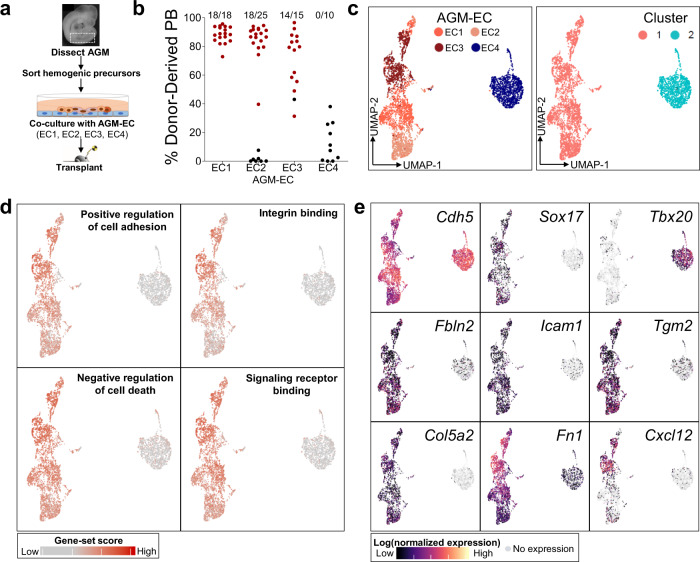
Single-cell RNA-sequencing identifies the transcriptional signatures of AGM-EC that differentially support HSC generation in vitro. **a** Methodology for comparison of AGM-EC in supporting the generation of engrafting HSC from E9-10 AGM/P-Sp-derived VE-cadherin^+^ hemogenic precursors. **b** Donor-derived peripheral blood (PB) engraftment in recipients transplanted with the progeny of hemogenic precursors following co-culture on various independently generated AGM-EC (EC1-4). The numbers above indicate the fraction of mice with multilineage engraftment, designated by data points in red. (PB engraftment shown at ≥24 weeks post transplant, pooled from *n* ≥ 3 independent experiments for each AGM-EC, from embryos in 18–35 somite pair range; source data are provided as a Source Data file). **c** UMAP and cluster analysis of single-cell transcriptional profiles of each AGM-EC from **b**. (See also Supplementary Fig. 1). **d** Gene-set scores representing top GO terms for transcripts differentially expressed by HSC-supportive verses non-supportive AGM-EC (biological processes and molecular functions ranked by *p* value, see also Supplementary Data 1, Supplementary Data 2). **e** Gene expression heatmap for pan-endothelial marker, VE-Cadherin (*Cdh5*), and selected transcripts differentially expressed between clusters. (See also Supplementary Data 1).

Identifying the microenvironmental signals necessary for the generation of functional HSCs requires analysis of niche cells capable of supporting the process of HSC specification and self-renewal. We previously demonstrated that Akt-activated AGM-derived EC (AGM-EC), which retain characteristics of their primary cells of origin including expression of Notch ligands, provides an instructive in vitro niche for the generation and expansion of HSCs from embryonic hemogenic precursors^27^. Most independently generated AGM-EC support the production of long-term, multilineage-engrafting HSCs following co-culture of AGM-derived VE-Cadherin^+^ hemogenic precursors isolated at E9-E10; however, we identified rare AGM-EC that are deficient in this capacity (Fig. 1a, b). To determine differences in transcriptional properties of HSC-supportive and non-supportive AGM-EC, we compared their genome-wide transcriptional profiles by scRNA-seq. Dimensionality reduction by Uniform Manifold Approximation (UMAP) and clustering of single-cell transcriptomes revealed that the non-supportive AGM-EC cluster apart from three independent HSC-supportive AGM-EC in transcriptional space (Fig. 1c, Supplementary Fig. 1). We then examined genes differentially expressed between the HSC-supportive and non-supportive AGM-EC, identifying genes most specific to the HSC-supportive cluster, which we hypothesize to include genes encoding signaling ligands essential for HSC generation (Supplementary Data 1). Gene ontology analysis of this gene-set suggests a prominent role in HSC-supportive AGM-EC for genes involved in the regulation of cell adhesion, integrin interactions, signaling receptor/growth factor binding, and cell death/apoptosis (Fig. 1d, Supplementary Data 2). Amongst the top significantly differentially expressed genes were transcription factors implicated in arterial EC and HSC fates, such as *Sox17*^7,32–34^, genes encoding secreted signaling ligands, such as the chemokine *Cxcl12*, and genes encoding cell adhesion and extracellular matrix proteins, particularly those interacting with integrins, such as *Icam1, Vcam1*, *Fn1* (Fibronectin), and *Tgm2* (Tissue transglutaminase) (Fig. 1e, Supplementary Data 1). These results identify candidate signals that may support the maturation and self-renewal of HSCs from hemogenic precursors in the AGM-EC niche, as well as transcription factors that may regulate the differential expression of niche signals between HSC-supportive and non-supportive AGM-EC.

### VE-Cadherin^+^CD61^+^EPCR^+^ surface phenotype enriches for functional HSC precursors during the transition from HE to HSC in the AGM

**Fig. 2:**
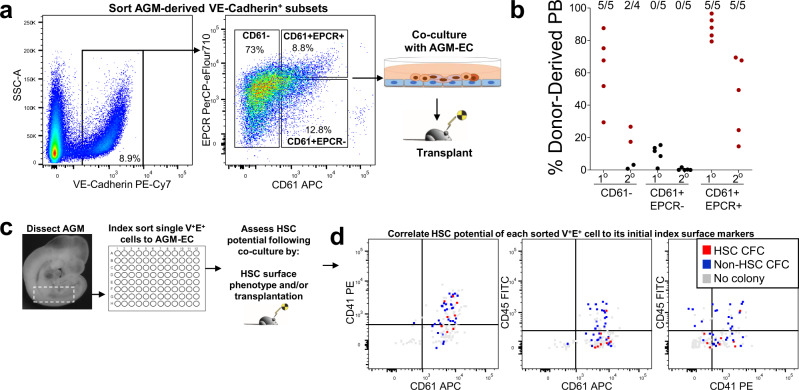
VE-Cadherin^+^CD61^+^EPCR^+^ (V^+^61^+^E^+^) surface phenotype enriches for functional HSC precursors during the transition from HE to HSC in the AGM. **a** Sorting by FACS of E9 (18-21 sp) AGM/P-Sp-derived cells based on the expression of CD61 and EPCR within the VE-Cadherin^+^ population (see also Supplementary Fig. 2). **b** Each population in **a** was co-cultured on AGM-EC, transplanted to lethally irradiated adult mice, and analyzed for donor-derived peripheral blood (PB) engraftment in primary (20 weeks) and secondary (16 weeks) recipients. The numbers above indicate the fraction of mice with multilineage engraftment in each group, designated by data points in red. (Similar results obtained in *n* = 1 independent experiment; source data are provided as a Source Data file). **c** Methodology for index sorting of single VE-Cadherin^+^EPCR^+^ (V^+^E^+^) cells for co-culture on AGM-EC and subsequent analysis to assess HSC potential by flow cytometry and transplantation. **d** Correlation of clonal HSC potential with surface expression of CD61, CD45, and CD41 on individual index-sorted V^+^E^+^ cells from E11 (40-42sp) AGM. HSC colony-forming cell (HSC CFC) (red) indicates the formation of a hematopoietic colony with HSC activity as detected by surface phenotype (VE-Cad^−/low^CD45^+^Gr1^−^F4/80^−^Sca1^hi^EPCR^hi^) and long-term (≥24 weeks) multilineage engraftment. (Source data are provided as a Source Data file). Non-HSC CFC (blue) indicates the formation of a hematopoietic colony lacking HSC phenotype and/or long-term engraftment. No colony (gray) indicates the absence of detectable hematopoietic cells following AGM-EC co-culture. (See also Supplementary Figs. 2–4).

Identifying signaling ligands from the transcriptomic analysis of AGM-EC that have functional relevance to HSC formation requires complementary knowledge of the cognate receptors and downstream pathways activated in hemogenic precursors during HSC specification and self-renewal. To capture this information, we next sought to characterize the single-cell transcriptomes of hemogenic precursors during their transition from HE to HSC. Given the rarity of HSCs and their precursors during early murine development, this requires further enrichment of HSC precursors independent of hematopoietic-specific markers such as CD41 and CD45 that would fail to capture HE^14,19^. Using the AGM-EC co-culture system to screen for surface markers that enrich for HSC precursor activity, we previously demonstrated that HSC precursors from E9 to E11 are primarily restricted to the subset of VE-Cadherin^+^ cells expressing EPCR (CD201), a marker of HSCs throughout development and in the adult^22,28,29,35,36^. We further determined that co-expression of EPCR with CD61, a surface marker expressed by HE^37^ and hematopoietic progenitors/HSC in the AGM^38^, defines the minor subset of total AGM-derived VE-Cadherin^+^ cells with the most robust long-term HSC potential, as measured by serial transplantation following AGM-EC co-culture of sorted populations from as early as E9 (Fig. 2a, b, Supplementary Fig. 2a). Within the VE-Cadherin^+^CD61^+^EPCR^+^ population (hereafter, referred to as V^+^61^+^E^+^) between E9 and E11, CD61 (Integrin-β3) is primarily co-expressed with co-receptor CD51 (Integrin-αv), while heterogeneous for co-receptor CD41 (Integrin-α2b), which is the earliest marker of hematopoietic specification from HE^17,38–40^ (Supplementary Fig. 2b, c, Supplementary Fig. 4a). Moreover, the V^+^61^+^E^+^ population at E10 to E11 co-expresses surface markers associated with HE/HSC potential, including CD133 (Prominin 1), AA4.1, ESAM, and CD105 (Endoglin), specifically enriches for expression of arterial markers CD44 and DLL4 (both recently associated with pre-HSC/HSC activity in the AGM^24,28,30,41^) within the AGM VE-Cadherin^+^ EC population, and heterogeneously express hematopoietic-specific markers CD41, CD43, CD45, and c-kit (Supplementary Fig. 2d). Immunostaining of E10.5 mouse embryo sections also confirmed that expression of EPCR is largely restricted to VE-Cadherin-expressing EC and intra-aortic hematopoietic clusters (IAHC) lining the aorta of the AGM (Supplementary Fig 3). Although immunostaining is unable to provide any direct correlation to functional HSC potential, cells co-expressing VE-Cadherin, CD61, and EPCR are identified heterogeneously within IAHC of the AGM, consistent with previous studies suggesting at least a subset of HSC precursors are contained within IAHC at this stage^19,41^ (Supplementary Fig 3c).

We previously reported a method utilizing single-cell index sorting of embryonic hemogenic precursors with AGM-EC co-culture followed by functional analysis in transplantation assays to characterize the phenotypic properties of clonal precursors with the capacity to give rise to colonies containing HSC (hereafter, referred to as HSC colony-forming cells, HSC CFC) and those giving rise to colonies lacking HSC potential (non-HSC CFC)^28,29^. Using this approach, we confirmed that the V^+^61^+^E^+^ population, though heterogeneous for hematopoietic potential at the clonal level, contains HSC CFC that significantly increased in frequency between E9 and E11, and encompasses HSC CFC heterogeneous for CD41 and CD45 expression, thus capturing HSC precursors at different stages of development during their transition from phenotypic HE (CD41^−^CD45^−^) to pro-HSC/pre-HSC I (CD41^+^CD45^−^) and pre-HSC II/HSC (CD45^+^)^16,17,42^ (Fig. 2c, d, Supplementary Fig. 4). Collectively, these results demonstrate that the V^+^61^+^E^+^ population, while heterogeneous for HSC potential at the single-cell level, significantly enriches for rare HSC precursors within the AGM across their developmental spectrum starting from the earliest subset defined phenotypically as HE.

### Single-cell RNA-sequencing of V^+^61^+^E^+^ cells identifies dynamic transcriptional signatures of HSC precursors during their transition from arterial-like HE in the AGM

**Fig. 3:**
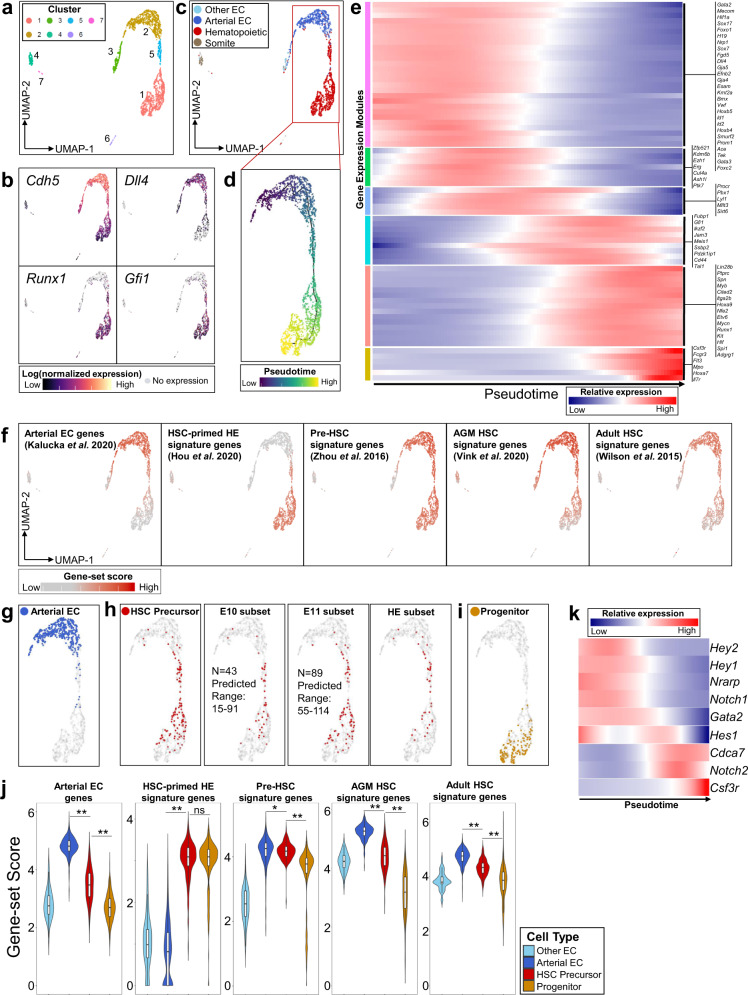
Single-cell RNA-sequencing of AGM V^+^61^+^E^+^ cells identifies dynamic transcriptional signatures of HSC precursors during their transition from arterial-like HE in vivo. **a** UMAP and cluster analysis of sorted E10 to E11 AGM-derived V^+^61^+^E^+^ cells. **b** Expression heatmap of representative pan-endothelial (*Cdh5*/VE-Cadherin*)*, arterial (*Dll4*), and hematopoietic-specific (*Runx1, Gfi1*) genes. **c** Cell type classification of single cells based on marker genes (See also Supplementary Fig. 5f). **d** Trajectory analysis using Monocle to order cells from adjacent clusters (1, 2, and 5) in pseudotime. **e** Gene expression heatmap of selected differentially expressed genes over pseudotime, segregated by modules of gene expression (See also Supplementary Data 3). **f** Heatmap of gene-set scores for signature genes defining arterial EC, HSC-primed HE, AGM pre-HSC, E11 AGM HSC, and adult bone marrow HSC, based on previously reported gene sets (see also Supplementary Figs. 6–7, Supplementary Data 6). **g**–**i** Cell type classification of single cells based on gene expression (see also Supplementary Fig. 5f): **g** Arterial EC (co-expressing *Cdh5, Dll4, Efnb2, Hey1*, not expressing *Runx1, Gifi1, Nr2f2, Nrp2*), **h** HSC precursor type (co-expressing *Gfi1, Mycn, Pdzk1ip1, Procr, Dll4, Vwf, Cdkn1c, Pbx1, Mllt3*, not expressing *Flt3, Il7r, Fcgr3, Csf3r*), including subset from E10 and E11 data (total numbers of transcriptionally defined HSC precursors and range predicted by functional HSC CFC assay at each stage are indicated, see also Supplementary Fig 4e), and a subset of HSC precursors transcriptionally defined as HE (lacking expression of *Itga2b*/CD41, *Spn*/CD43, and *Ptprc*/CD45). **i** Progenitor cell type (expressing *Runx1* plus one or more of *Flt3, Il7r, Fcgr3*, or *Csf3r*). **j** Gene-set scores for signature genes from (f) in transcriptionally defined cell types (boxplots show median values and interquartile ranges; upper/lower whiskers show 1.5× interquartile range, *p = 0.026; **p < 1E-15; ns *p* = 0.76, unpaired, two-sided Wilcoxon test). **k** Gene expression heatmap of Notch receptors and target genes in pseudotime.

To study the transcriptional profiles of HSC precursors during their emergence from HE, we next isolated V^+^61^+^E^+^ cells by fluorescence-activated cell sorting (FACS) from dissected AGM and performed scRNA-seq. Given HSC precursors are exceedingly rare prior to E10, we focused on AGM dissected between E10 and E11, during the peak emergence of HSC precursors from HE based on our data (Supplementary Fig 4e) and previously published studies^14,28^. We obtained scRNA-seq data from 3092 cells isolated from a total of 100 embryos pooled in two independent experiments at E10 (31–39 somite pairs) and E11 (40–45 somite pairs) (Supplementary Fig. 5a–e). Dimensionality reduction and unsupervised clustering of the single-cell transcriptomes showed that most cells reside in a series of adjacent clusters (1, 2, and 5) differentially expressing endothelial markers (*Cdh5*, encoding VE-cadherin, and *Dll4*, encoding Notch ligand Delta-like-4, expressed in arterial EC) and hematopoietic fate-specifying transcription factors (*Runx1* and *Gfi1*), consistent with cells undergoing a transition from an endothelial to hematopoietic transcriptional program (Fig. 3a, b). Cell type classification based on marker genes identified that the major clusters (1, 2, and 5) are comprised of cells primarily expressing markers of arterial EC and hematopoietic fates (Fig. 3c, Supplementary Fig. 5f). Several smaller clusters segregated in transcriptional space express markers of differentiated myeloid and erythroid/megakaryocyte lineages (cluster 6), non-arterial EC (cluster 3), and somitic mesoderm (cluster 4), likely representing minor contaminating populations following FACS, which were excluded for subsequent analyses (Fig. 3c, Supplementary Fig. 5g).

We then applied an unbiased machine learning algorithm using Monocle^43^ to generate a trajectory by pseudotemporal ordering of single cells (Fig. 3d) and to identify genes that vary significantly across pseudotime (Supplementary Data 3). Gene expression changes over pseudotime suggest this trajectory recapitulates the developmental emergence of pre-HSC/HSC (enriched in HSC-associated genes, e.g., *Pdzk1ip1, Hlf, Etv6, Mycn*) from an initial population expressing genes consistent with arterial EC/HE transcriptional programs (eg. *Dll4*, *Gja5*, *Nrp1*, *Sox17*, *Foxc2*, *Gata2*) (Fig. 3e) and genes associated with HSC self-renewal (e.g*., Erg, Kdm6b, Mllt3, Mecom, Pbx1, Sirt6*)^44–49^. Cells at the terminal portion of the pseudotemporal trajectory demonstrate changes in gene expression consistent with loss of HSC potential and acquisition of lymphoid/myeloid progenitor fates (e.g., expression of *Flt3, IL7r, Csf3r*, *Fcgr3*) (Fig. 3e).

Given that the V^+^61^+^E^+^ population is heterogeneous for cells with clonal HSC potential (HSC CFC, Fig. 2d, Supplementary Fig 4), we next utilized an in silico approach to further resolve putative HSC precursors in our data. To characterize gene expression changes associated with HSC development, we calculated aggregated gene-set scores using published arterial EC marker genes^50^ and genes that have been identified as specific to HSC-primed HE^26^, AGM pre-HSCs^22^, E11 AGM HSCs^30^, and adult bone marrow HSCs^31^ in previous studies using scRNA-seq data (Fig. 3f, Supplementary Figs. 6, 7, Supplementary Data 6). Whereas the HSC-primed HE gene-set was defined by genes differentially expressed between non-hemogenic arterial EC and HE^26^, the AGM pre-HSC/HSC gene sets distinguished pre-HSC/HSC from more differentiated hematopoietic progenitors^22,30^, thus predicting that cells encompassing the HE to pre-HSC/HSC transition should express overlapping genes contained within each of these signature gene sets. Based on this prediction, we defined a combined set of genes representing each of these gene sets that have also been previously validated as pre-HSC/HSC markers or are functionally required for HSC development and self-renewal (*Gfi1, Mycn, Pdzk1ip1, Procr, Dll4, Vwf, Cdkn1c, Pbx1, Mllt3*)^22,28–30,35,45,48,51–54^. We then applied an in silico approach to further define an “HSC precursor” cell type based on co-expression in single cells of these nine genes and absence of expression of progenitor-specific genes (*Flt3, IL7r, Csf3r*, and *Fcgr3*) (Fig. 3h)^52,55^. As predicted, the transcriptionally defined “HSC precursors” are contained in an intermediate portion of pseudotime enriched with cells with high gene-set scores for both HSC-primed HE and pre-HSC/HSC gene sets (Fig. 3f, h, j). Furthermore, “HSC precursors” comprise a comparable number of cells in the V^+^61^+^E^+^ scRNA-seq data to that predicted based on HSC CFC determined functionally by single-cell index and co-culture analysis at E10 and E11 (Fig. 3h, Supplementary Fig. 4e)^14^ and include a transcriptionally defined subset of HE that lack expression of hematopoietic markers (*Itga2b*/CD41, *Spn*/CD43, and *Ptprc*/CD45) (Fig. 3h). We also defined a “progenitor” cell type based on co-expression of *Runx1* together with one or more progenitor-specific genes *Flt3, IL7r, Csf3r*, or *Fcgr3*; expectedly, cells of this type were largely confined to the terminal portion of pseudotime (Fig. 3i) and demonstrated lower arterial EC, AGM pre-HSC, and HSC gene-set scores than “HSC precursor” cell types (Fig. 3j). Altogether, our single-cell transcriptional data, comprising a population enriched for functionally validated HSC precursors, combined with further in silico analysis, successfully captures the developmental emergence of HSCs from HE, which is uniquely characterized by overlapping arterial endothelial and HSC-specific transcriptional signatures.

By distinguishing the transcriptional profiles of the rare transitional population of emerging HSC precursors from more differentiated hematopoietic progenitors, this analysis enables us to identify receptors and downstream signal pathways specifically expressed during pre-HSC/HSC development from HE. For example, to study the dynamics of Notch signaling during this process, we examined the relative expression of genes activated by the Notch pathway over pseudotime (Fig. 3k). During the maturation of HE to pre-HSC/HSC, concomitant with decreasing *Notch1* and increasing *Notch2* expression, expression of Notch target genes *Hey1/Hey2*, required for arterial EC fates^41^, are downregulated, and Notch target gene *Cdca7*, which regulates HSC emergence^56^, is increased. Notch targets *Gata2* and *Hes1*, also essential for HSC development^57–59^, are sequentially downregulated in the terminal portion of pseudotime corresponding with increased expression of genes specific to differentiation to progenitors (*Csf3r*). This pattern of Notch pathway gene expression changes is consistent with recent studies suggesting that, although Notch1 is required early for definitive hematopoietic fate, the emergence of HSCs from HE correlates with decreased *Notch1* expression, lower overall Notch signal strength, and a requirement for *Hes1* in limiting Notch-dependent *Gata2* expression^6,59–62^. These results demonstrate the utility of our single-cell transcriptomic analysis to elucidate the developmental dynamics of signaling pathways during the HE to pre-HSC/HSC transition.

### Single-cell RNA-sequencing identifies the transcriptional signatures of self-renewing HSCs emerging from AGM V^+^61^+^E^+^ precursors in vitro

**Fig. 4:**
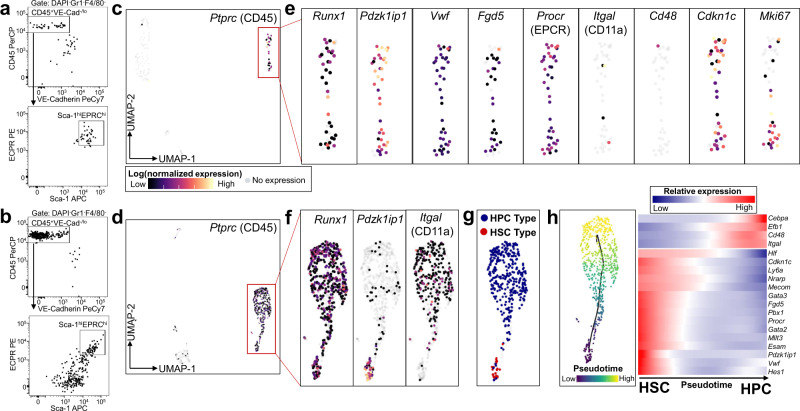
Single-cell RNA-sequencing identifies the transcriptional signatures of self-renewing HSCs emerging from clonal V^+^61^+^E^+^ hemogenic precursors in the AGM-EC niche in vitro. **a**, **b** FACS analysis of the progeny of a single V^+^61^+^E^+^ hemogenic precursor from E11 AGM (42sp) following 4-day co-culture on AGM-EC, with **a** HSC phenotype (VE-Cad^low/−^CD45^+^Gr1^−^F4/80^−^Sca1^hi^EPCR^hi^) and **b** mixed HSC and hematopoietic progenitor cell (HPC) phenotype. **c**, **d** UMAP projection of single-cell transcriptomes obtained from cells from **a**, **b** showing hematopoietic cluster (red box) expressing pan-hematopoietic marker *Ptprc* (CD45). **e** Expression of *Runx1*, HSC marker genes (*Pdzk1ip1, Vwf, Procr, Fgd5*), HPC-associated genes (*Itgal, Cd48*), and cell cycle genes (*Cdkn1c*, *Mki67*) in the hematopoietic cluster from **c**. **f** Expression of *Runx1*, *Pdzk1ip1, and Itgal* in the hematopoietic cluster from **d**. **g** Cell type classification of hematopoietic cells based on gene expression: HSC Type (expressing *Runx1, Pdzk1ip1*, and *Vwf*, not expressing *Cd48* or *Itgal*) and HPC type (expressing *Cd48* and/or *Itgal*). **h** Trajectory analysis using Monocle to order cells from the hematopoietic cluster in pseudotime, and gene expression heatmap over pseudotime of selected genes, including Notch pathway targets, differentially expressed during HSC differentiation to HPC. (See also Supplementary Fig. 8, Supplementary Data 4).

We previously showed that a single AGM-derived hemogenic precursor could give rise to more than one hundred long-term engrafting HSCs following AGM-EC co-culture, suggesting that the AGM-EC niche provides signals sufficient to support both the maturation and self-renewal of HSCs^28^. Therefore, we hypothesized that single-cell transcriptomic analysis of HSCs generated during AGM-EC co-culture would identify receptors and downstream signal pathways essential to HSC formation and self-renewal in vitro, complementing our analysis of primary HSC precursors undergoing the initial transition from HE to HSC in the AGM in vivo. Thus, we next sought to acquire the transcriptional profiles of HSCs emerging from embryonic hemogenic precursors in the AGM-EC vascular niche by performing scRNA-seq on the progeny of individual FACS-isolated E11 AGM-derived V^+^61^+^E^+^ cells during colony formation in AGM-EC co-culture. Cells were harvested at day 4 of co-culture, and 50% of the cells were analyzed for surface phenotype we previously showed to correlate with long-term engrafting HSC activity (VE-Cadherin^−/low^CD45^+^Gr1^−^F4/80^−^Sca1^hi^EPCR^hi^)^28,29^. scRNA-seq was performed from the remaining cells from two colony types, one with a relatively homogeneous population of phenotypic HSCs (Fig. 4a) and another consisting of a mixed population of phenotypic HSCs and cells with decreasing expression of Sca1 and EPCR consistent with differentiation to hematopoietic progenitor cells (HPC) (Fig. 4b). Dimensionality reduction and clustering analysis identified distinct clusters consistent with hematopoietic progeny (expressing *Ptprc/*CD45) for each colony type (Fig. 4c, d, Supplementary Fig. 8). Consistent with their HSC phenotype, the cells from the hematopoietic cluster in the first colony type uniformly expressed established HSC marker genes (such as *Pdzk1ip1*, *Procr, Fdg5, Vwf*)^35,52,54,63^ and largely lacked expression of genes associated with differentiation of HSC to HPC (*Itgal/*CD11a*, Cd48*)^64,65^ (Fig. 4e). Consistent with a profile of self-renewing HSCs, cells in this colony type expressed HSC-associated cell cycle regulator *Cdkn1c* (p57) and a subset expressed active cell cycle gene *Mki67* (Fig. 4e). Hematopoietic cells in the second colony type demonstrated a similar, minor population of HSCs based on their transcriptional signatures (“HSC Type”, co-expressing *Runx1*, *Pdzk1ip1,* and *Vwf*, negative for *Cd48* and *Itgal*) and a larger population of emerging HPC (“HPC Type”, expressing *Cd48* and/or *Itgal*) (Fig. 4f, g, Supplementary Fig. 8e–h). Pseudotemporal ordering recapitulated the differentiation of HPC from HSC, revealing gene expression changes during this process, including increased expression over pseudotime of genes involved in early myeloid and lymphoid-specific fates, e.g., *Cebpa* and *Ebf1* (Fig. 4h, Supplementary Data 4). Consistent with our previous studies showing that both emergence of HSCs from HE and expansion of HSCs in the vascular niche is Notch-dependent^27,66^, analysis of scRNA-seq data from HSCs generated during co-culture with AGM-EC demonstrates that Notch target genes required for HSCs (*Hes1, Gata2*) are enriched in cells in early pseudotime expressing HSC-specific programs, including genes involved in HSC self-renewal, such as *Cdkn1c*, *Mecom*, *Pbx1*, and *Mllt3*, with downregulation concomitant with differentiation to HPC (Fig. 4h). Collectively, this scRNA-seq data captures the transcriptomes of HSCs during their emergence from clonal V^+^61^+^E^+^ precursors and reveals the dynamics of HSC self-renewal and early HSC differentiation to HPC in the AGM-EC niche.

### Complementary analysis of the transcriptional profiles of AGM-EC and developing HSCs identify ligand–receptor interactions supporting HSC development

**Fig. 5:**
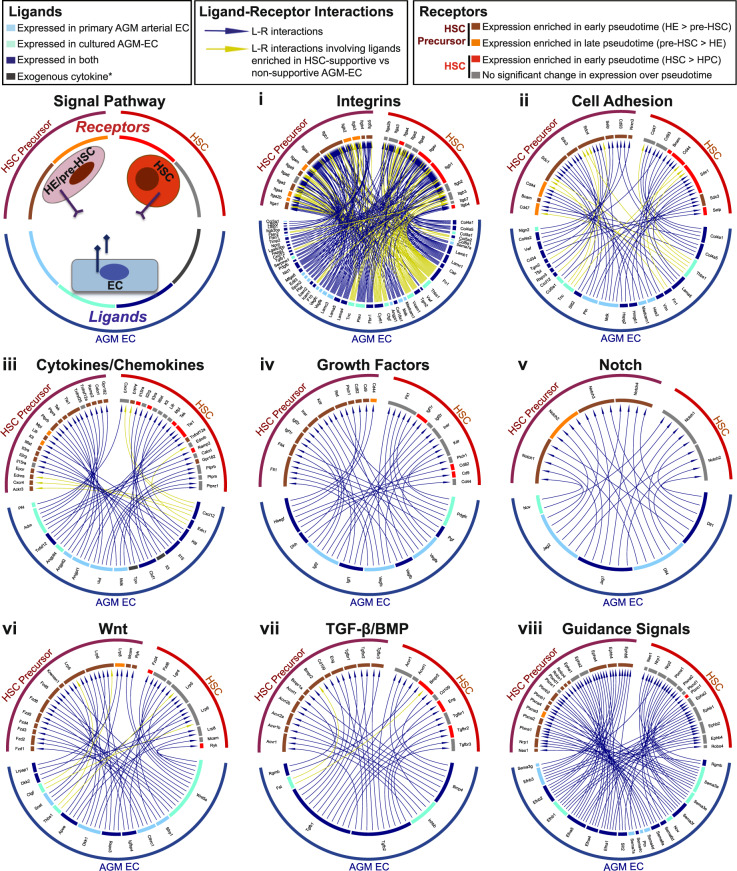
Signaling interactions identified by ligand–receptor analysis. Selected signal pathways regulated by ligand–receptor interactions between AGM niche EC and developing HSCs based on complementary scRNA-seq analysis. HSC Precursor: Indicates receptors expressed in primary AGM V^+^61^+^E^+^ cells classified as “HSC Precursor” types (see Fig. 3h). HSC: Indicates receptors expressed in cells generated following AGM-EC co-culture of V^+^61^+^E^+^ cells classified as “HSC” type (see Fig. 4g, Supplementary Fig 8f–h). Receptors were also classified as to whether they were expressed more highly in early (enriched in HE) or late (enriched in pre-HSC) pseudotime for primary AGM V^+^61^+^E^+^ cells, or expressed more highly in early pseudotime (enriched in HSCs vs HPCs) in cells generated following AGM-EC co-culture. Ligands were classified by whether they were expressed in HSC-supportive AGM-EC (cultured AGM-EC), primary AGM-derived AGM V^+^61^+^E^+^ cells classified as “arterial EC” (primary AGM arterial EC), or both. Signals mediated by ligands differentially expressed in HSC-supportive vs non-supportive AGM-EC are indicated by yellow arrows (see Supplementary Data 1). *indicates exogenous cytokines in AGM-EC culture conditions. (See also Supplementary Data 5 for a complete list of identified ligands, receptors, and ligand–receptor interactions).

For the AGM-EC niche to support the generation of functional HSCs from HE, it must express a repertoire of signaling ligands necessary to promote both the maturation and self-renewal of HSC. In parallel, developing HSCs must express the cognate receptors required to receive these essential niche-derived signals. Thus, we hypothesized that analysis of the complementary transcriptomes of AGM niche EC and HSC precursors transitioning to self-renewing HSCs from our in vivo and in vitro scRNA-seq data could be used to identify the ligand–receptor interactions necessary to support HSC development. To test this, we applied a comprehensive database consisting of curated pairs of ligands and receptors^67^ to our scRNA-seq data to infer potential functionally relevant interactions. We first identified ligands expressed in the HSC-supportive AGM-EC (from Fig. 1), as well as primary AGM-derived arterial EC (“Arterial EC” Type, from Fig. 3c–g), which share common phenotypic characteristics and overlapping gene expression profiles (Supplementary Fig. 9a–c). We then identified receptors expressed in the subset of AGM-derived V^+^61^+^E^+^ cells transcriptionally defined as “HSC precursor” type (from Fig. 3h), representing cells encompassing the transition from HE to pre-HSC/HSC. These data sets were then combined to determine the global set of ligand–receptor interactions between AGM niche EC and HSC precursors (Fig. 5, Supplementary Data 5). We also identified receptors expressed in cells defined by “HSC Type” emerging during AGM-EC co-culture of AGM-derived V^+^61^+^E^+^ cells (from Fig. 4g, Supplementary Fig. 8f, g) and combined this data with AGM niche EC ligands to determine the global set of ligand–receptor interactions potentially regulating HSC maturation and self-renewal (representative interactions organized by pathways shown in Fig. 5, comprehensive list in Supplementary Data 5). We used an unbiased approach to identify ligands and receptors based only on expression thresholds in each cell type, so as to provide a comprehensive list of potential interactions (see Methods for further details). Validating the approach, this integrated analysis identified receptor-ligand interactions modulating signaling pathways with established functions in HSC development and self-renewal, including Notch and Wnt^5,68–70^, BMP^34^, Hedgehog^71^, Ephrins^72^, CD44^24^, P-selectin^73^, Pleiotrophin^74^, Angiopoietin-like proteins^75^, chemokines^76,77^, IGFs^78,79^, and vasoactive factors whose roles in HSC development were recently published, such as endothelin-1^80^ and adrenomedullin^81^. The unbiased analysis also identified potentially novel signaling interactions and modulators of key pathways that have not previously been described to have a specific role in HSC development, including numerous extracellular matrix proteins interacting with integrins and cell adhesion receptors, cytokines/chemokines, growth factors, including signaling proteins that are both positive and negative regulators of canonical/non-canonical Wnt and TGF-β/BMP pathways, and guidance signal pathways such as semaphorins (Fig. 5i–viii, Supplementary Data 5).

### Functional analysis of ligand–receptor interactions enables the engineering of an in vitro niche supporting HSC development from hemogenic precursors

Building from the ligand–receptor interactions identified by our single-cell transcriptomic analysis, we next sought to engineer an artificial stroma-independent niche sufficient to recapitulate the function of AGM-EC in supporting the generation of long-term engrafting HSCs from embryonic HSC precursors. We previously identified a set of conditions incorporating immobilized Notch ligand Delta1 (Dll1-Fc) that was sufficient to support the quantitative expansion of HSCs isolated from E11 AGM, at which time in development engrafting HSCs are already detectable at low frequency by direct transplantation into adult recipients^27^. However, these conditions were unable to support the generation of engrafting HSCs from hemogenic precursors isolated prior to E11. We adapted these conditions by incorporating serum-free media to further control for exogenous factors. Based on cytokine receptor expression profiles in the scRNA-seq data in AGM-derived HSC precursors and in vitro generated HSCs (Fig. 5iii, Supplementary Data 5), we retained recombinant stem cell factor (SCF), interleukin-3 (IL-3), and thrombopoietin (TPO). Based on our single-cell transcriptomic analysis and reported studies suggesting dynamic roles for Notch1 and Notch2 receptors in activating downstream Notch targets expressed during pre-HSC formation from HE and subsequent HSC maturation and self-renewal^6,27,60–62,82,83^ (Fig. 3k, Fig. 4h, Fig. 5v), we also incorporated immobilized anti-Notch1 and anti-Notch2 monoclonal antibodies in place of immobilized Dll1-Fc. These antibodies specifically bind Notch1 and Notch2 receptors, respectively^27^, enabling targeted activation of Notch1 and/or Notch2 receptors independent of Fringe-mediated modifications that could alter their binding and activation by ligands such as Dll1^84^.

Genes involved in integrin-binding/cell adhesion were among those most differentially expressed in HSC-supportive verses non-supportive AGM-EC, particularly multiple integrin ligands associated with VLA-4 interactions, including *Fn1* (fibronectin) and *Vcam1* (Fig. 1d, Supplementary Fig. 9b, c, Supplementary Data 1). We validated VLA-4/CD49d (*Itga4*) surface protein expression, which was identified by scRNA-seq analysis on HSC precursors (Fig. 5i, Supplementary Data 5), by flow cytometry on AGM-derived V^+^61^+^E^+^ cells, and by index analysis of cells with HSC CFC potential (Supplementary Figure 10a–e). Based on these findings suggesting a role for ligand–receptor integrin interactions via VLA-4 in the AGM-EC niche, we also utilized immobilized recombinant fibronectin fragment (FN-CH-296), which specifically binds cell-surface VLA-4 and VLA-5 in vitro.

**Fig. 6:**
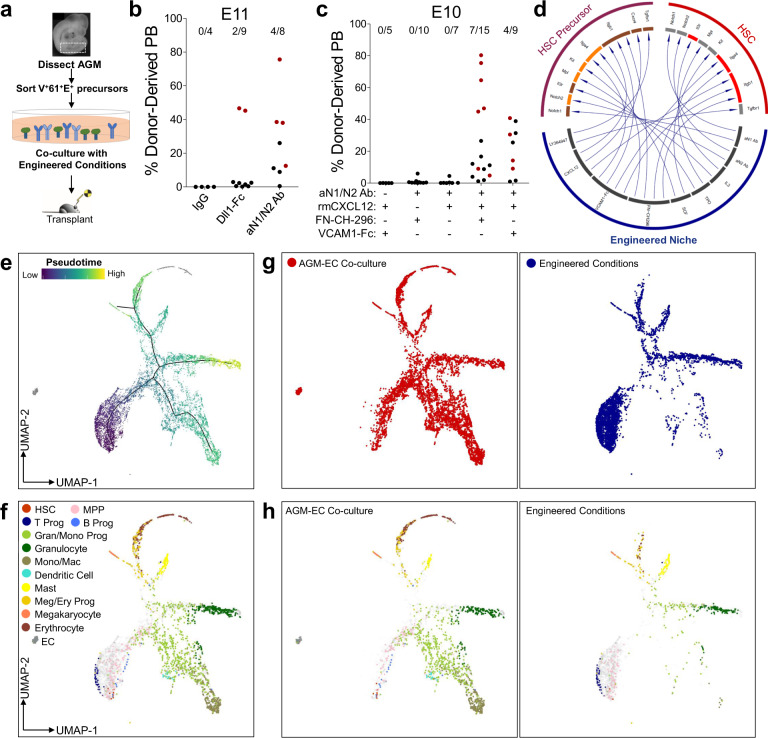
Functional validation of ligand–receptor interactions enables the engineering of an in vitro niche supporting HSC development. **a** Methodology evaluating generation of engrafting HSC from AGM-derived V^+^61^+^E^+^ hemogenic precursors in engineered conditions. **b** Donor-derived peripheral blood engraftment (≥24 weeks) in recipients transplanted with the progeny of E11 AGM-derived V^+^61^+^E^+^ hemogenic precursor following co-culture in engineered conditions with immobilized Dll1-Fc, anti-Notch1/Notch2 (aN1/N2 Ab), or control (IgG). Data points in red indicate mice with multilineage engraftment (results pooled from *n* = 2 independent experiments). (See also Supplementary Fig. 11. Source data are provided as a Source Data file). **c** Donor-derived peripheral blood engraftment (≥24 weeks) in recipients transplanted with the progeny of E10 AGM-derived V^+^61^+^E^+^ hemogenic precursor following co-culture in engineered conditions as indicated (results pooled from *n* = 5 independent experiments). (Source data are provided as a Source Data file). **d** Ligand–receptor interactions recapitulated in the engineered niche. **e** UMAP and pseudotemporal ordering of single-cell transcriptomes of the progeny of E10 V^+^61^+^E^+^ hemogenic precursors following 6 days of culture on AGM-EC or engineered conditions. **f** Cell type classification based on marker genes. Undefined cell types are shown in gray. **g**, **h** Subsets of all single cells (**g**) and cell types (**h**) derived from cells cultured on AGM-EC (left panels) or engineered conditions (right panels). (See Supplementary Fig. 11).

We then tested whether components of this engineered niche including immobilized FN-CH-296, cytokines (IL-3, SCF, and TPO), and small-molecule inhibitor of TGF-β receptor (LY364947), and either immobilized Dll1-Fc, anti-N1, and anti-N2 antibodies (aN1/N2 Ab), or IgG (control), could support in vitro generation of HSCs. Long-term engrafting HSCs were detected following culture of E11 AGM-derived V^+^61^+^E^+^ cells in engineered conditions with either immobilized Dll1-Fc or combined aN1/N2 antibodies, but not control IgG (Fig. 6a, b, Supplementary Fig. 11a–c). However, these conditions were unable to support the generation of engrafting HSCs from V^+^61^+^E^+^ cells isolated from AGM prior to E11 (Fig. 6c), suggesting additional signals are required to promote the maturation of HSCs from hemogenic precursors at earlier stages.

We hypothesized that signaling interactions required for HSC maturation from these earlier stages would involve receptors in HSC precursors whose expression is enriched in early pseudotime (i.e., greater in HE than in maturing pre-HSC and progenitors), and cognate ligands whose expression is enriched in HSC-supportive AGM-EC (which can promote HSC formation from E9-E10 haemogenic precursors) and also detected in primary AGM-derived EC (which serve as a niche for HSC formation from HE in vivo). Thus, we prioritized for further analysis ligand–receptor interactions that fit these criteria (Fig. 5, receptors indicated in dark red, ligands indicated in navy, ligand–receptor interactions indicated by yellow arrows). This identified genes encoding cell adhesion and extracellular matrix proteins that interact with integrin receptors, including *Col5a2* (a type V collagen), *Fn1* (fibronectin), and *Tgm2* (tissue transglutaminase, which can enhance integrin β1 interaction with fibronectin)^85^, and genes encoding secreted factors *Cyr61*, an integrin-interacting protein, *Edn1* (endothelin-1), a vasoactive peptide, and *Cxcl12*, a chemokine that interacts with the receptor encoded by *Cxcr4* expressed in HE based on our scRNA-seq data (Fig. 5iii, Supplementary Data 5). Supporting the transcriptional data, we found that rare AGM-derived V^+^61^+^E^+^ HSC CFC between E9 and early E10 expressed the CXCL12 receptor CXCR4, whereas CXCR4 expression is low or negative in most HSC CFCs by late E10 (Supplementary Fig. 10c, d)^86^. Consistent with a role for CXCL12/CXCR4 signaling supporting HSC development from hemogenic precursors at earlier stages, we found that the addition of recombinant CXCL12 enabled the generation of multilineage long-term engrafting HSCs from E10 AGM-derived V^+^61^+^E^+^ cells following in vitro culture (Fig. 6c), and in some experiments, also enabled the generation of HSCs from embryos as early as E9, though with lower efficiency than at E10 (see Source Data). Generation of HSCs in the presence of CXCL12 before E11 was also dependent on Notch activation by immobilized aN1/N2 Ab and the presence of a ligand for integrin VLA-4, with immobilized VCAM1-Fc interchangeable with fibronectin fragment (FN-CH-296) (Fig. 6c). Altogether, this approach successfully identified a group of signaling ligands that, when integrated into an in vitro engineered niche, was sufficient to support functional HSC generation from embryonic hemogenic precursors.

In addition to supporting the initial generation and self-renewal of HSCs from hemogenic precursors, we previously showed that prolonged co-culture in the AGM-EC niche supports multilineage hematopoiesis in vitro^27^. To compare the emergence of HSCs and downstream hematopoietic lineages in the engineered niche with AGM-EC co-culture, we performed scRNA-seq on the hematopoietic progeny generated following extended (6 days) culture of AGM-derived V^+^61^+^E^+^ cells in each condition. Hematopoietic progeny following culture in the engineered niche included rare cells with transcriptional signatures of HSC and multipotent progenitors, as expected, as well as cells differentiating to myeloid, erythroid/megakaryocytic, and lymphoid lineages (Fig. 6e–h, Supplementary Fig. 11d, e). Relative skewing of contribution toward early T lymphoid fate and away from B lymphoid, monocyte/macrophage, and late erythroid fates in engineered conditions suggests relatively increased activation of Notch pathway and/or deficiency of yet undefined signals presented by AGM-EC stroma. Collectively, these results demonstrate that a stroma-independent engineered niche is sufficient to partially recapitulate the vascular niche supporting HSC development from embryonic hemogenic precursors, and that further optimization through the incorporation of additional niche-derived signals identified by the ligand–receptor analysis and temporal modulation of key signal pathways such as Notch during culture could further enhance the balance of HSC generation and self-renewal versus lineage differentiation.

## Discussion

In this study, we provide several advances towards deciphering the complex signaling interactions required to support the specification and self-renewal of HSCs during embryonic development. First, using an ex vivo vascular niche modeling the embryonic AGM, where the first HSCs originate from HE, we identified a combination of phenotypic markers (V^+^61^+^E^+^) that enable enrichment of functionally validated HSC precursors encompassing the transition from HE to pre-HSC/HSCs. Second, using scRNA-seq of the primary AGM V^+^61^+^E^+^ population and the progeny of these cells during their maturation to HSCs and self-renewal in the AGM-EC niche, combined with computational analysis to analyze arterial EC and pre-HSC/HSC-specific gene expression changes over pseudotime, we generated a comprehensive transcriptional map of HSC development from HE. Third, we utilized these data to identify receptors and signal pathways expressed during HSC specification and self-renewal and, combined with complementary transcriptional analysis of the HSC-supportive AGM-EC niche to identify cognate ligands, produced an atlas of potential ligand–receptor interactions regulating HSC development in the vascular niche. Finally, we applied this knowledge to rationally design a stromal cell-independent engineered niche sufficient to generate engrafting HSC from embryonic hemogenic precursors in vitro.

Our approach to the single-cell transcriptomic analysis of HSC development builds on recently reported studies^21–24,26,30^, integrating overlapping HSC-specific gene signatures from published data sets of arterial EC, HE, pre-HSC, and HSC throughout development to refine the transcriptional signature defining HSC emergence from HE, and adding critical information about interactions with niche ECs supporting the process of HSC specification and self-renewal. Because we functionally validated HSC precursor activity within the V^+^61^+^E^+^ population independent of CD41 and CD45 expression, we were able to capture the transcriptional profiles of V^+^61^+^E^+^CD41^−^CD45^−^ HE that precede the emergence of V^+^61^+^E^+^CD41^+^CD45^+/−^ pre-HSC. Our functional single-cell analysis demonstrates, however, that the V^+^61^+^E^+^ population is heterogeneous for HSC potential at the single-cell level. Thus, further in silico analysis of scRNA-seq data from AGM-derived V^+^61^+^E^+^ cells was necessary to identify putative HSC precursors, which numerically correlated to HSC precursors detected by our functional assays, validating this approach. Altogether, combining functional, phenotypic, and transcriptional studies at the single-cell level, our analysis thus provides strong evidence that pre-HSC emergence from HE in the AGM is uniquely characterized by overlapping arterial endothelial and HSC-specific transcriptional programs, supporting studies from our group and others that suggest HSC potential resides within cells with arterial endothelial phenotypes^8,10,28^. Our studies thus suggest that recapitulating the conditions necessary to promote these arterial endothelial/HE programs in vitro will be a critical initial step to establishing methods for generating HSCs from pluripotent stem cells. Further supporting this concept, an elegant study of HSC development combining scRNA-seq with single-cell ATAC-seq, reported during the preparation of this manuscript, identified a population of putative precursors to HE characterized by high expression of arterial genes such as *Sox17* and *Hey2*, in which multiple hematopoietic stem and progenitor cell-associated transcriptional programs appear to converge to regulate *Runx1* expression^21^.

Our study also leveraged an AGM-derived EC niche model, which we previously showed could support the generation and expansion of long-term engrafting HSCs from embryonic precursors^27–29^, to identify niche-derived ligands essential to HSC specification and self-renewal. We were able to identify signaling ligands with functional relevance based on their differential expression in AGM-EC that varied in their ability to support HSC generation from hemogenic precursors prior to E11. We hypothesize that heterogeneity in the supportive properties of AGM-EC stroma may result from founder effects due to the small population of AGM-derived EC that initially survives following FACS and cell culture to establish each line. Previous studies suggest that mesodermal origin and dorsoventral positioning contribute to heterogeneity in the HSC-inductive properties of EC of the AGM aorta^77,87,88^. Interestingly, the non-supportive AGM-EC uniquely expressed *Tbx20*, a transcription factor previously shown to be expressed in endothelial cells localized to the dorsal portion of the embryonic aorta which lacks HSC activity (Fig. 1d)^34,87^. In contrast, HSC-supportive AGM-EC shared expression of *Sox17*, a transcription factor required for arterial EC/HE fates, with primary arterial EC and HE (Fig. 1, Supplementary Fig. 9)^7,33^. We hypothesize that V^+^61^+^E^+^ cells include non-hemogenic arterial ECs that deliver HSC-supportive signals to nearby V^+^61^+^E^+^ HE in the AGM. Supporting this hypothesis, primary AGM-derived V^+^61^+^E^+^ cells identified transcriptionally as arterial EC (Fig. 3c–f) exhibit a gene expression profile overlapping with that of HSC-supportive AGM-EC, including expression of essential niche ligands functionally validated in our engineered niche, such as *Cxcl12* and *Fn1* (Supplementary Fig. 9b, c). Future studies will further determine how differential HSC-supportive properties of AGM-EC may relate to the heterogeneity of the primary niche EC of the aorta from which they are derived, including such variables as differential expression of key transcriptions factors, mesodermal origin, dorsal/ventral position, the embryonic stage, and endothelial maturation or activation state.

By prioritizing ligand–receptor interactions that were specifically identified in HSC-supportive AGM-EC, our analysis highlighted a key role of integrins in HSC formation. Although integrin interactions are known to mediate aspects of HSC homing, retention, and maintenance in fetal liver and adult bone marrow niches, a study in zebrafish also demonstrated an important role for fibronectin-integrin β1 interactions in promoting Notch-mediated HSC fate in the embryonic aorta^89^. Consistent with this study, we determined that the combination of Notch activation, mediated by immobilized Notch1 and Notch2-receptor-specific antibodies, and a VLA-4 integrin (*Itga4*/*Itgb1*) ligand, either VCAM1-Fc chimera or Fibronectin fragment, was necessary to support HSC generation from embryonic AGM-derived hemogenic precursors in our engineered niche. Moreover, we identified a specific requirement for the chemokine CXCL12 in the engineered niche to enable the generation of engrafting HSCs from hemogenic precursors isolated prior to E11, the earliest developmental timepoint in which HSC activity is consistently detected by direct transplantation. The timing of this requirement for CXCL12 coincides with the detection of surface expression of its receptor CXCR4 on HSC precursors between E9 and E10, which is subsequently decreased by late E10, consistent with single-cell transcriptional analysis, which suggests *Cxcr4* expression in HE is downregulated during the transition to pre-HSC by pseudotemporal ordering. These results suggest a role for the chemokine CXCL12 in promoting HSC fate from less mature hemogenic precursors, distinct from the established role of the CXCL12/CXCR4 axis in homing/retention and maintenance of quiescence of adult HSCs in the bone marrow niche^90,91^. Consistent with our results, studies in zebrafish demonstrated that CXCR4 expression is induced in HE downstream of somite-derived retinoic acid signaling^92^ and that a subset of non-hemogenic aortic endothelial cells derived from somitic mesoderm supported HSC fate from aortic HE via production of CXCL12^77^. Although a comparable role for CXCL12/CXCR4 interactions during HSC formation from HE in the mammalian AGM has not yet been established to our knowledge, studies using murine and human pluripotent stem cells have demonstrated that CXCR4 is a marker of arterial HE enriched in lymphoid potential in vitro^93^, and that CXCL12 can promote multilineage hematopoietic output downstream of the hemangioblast stage^94^ and negatively regulate the endothelial potential of CXCR4-expressing HE^95^. Interestingly, VLA-4 integrin has also been shown to be activated by CXCL12 either downstream of CXCR4 interactions or by direct binding, potentiating its interactions with ligand VCAM1, suggesting an additional mechanism for CXCL12 enhancement of HSC generation in our engineered niche^96–98^. Although our studies suggest an important function for CXCL12 in the engineered niched during the initial HE to HSC transition, future studies will be required to determine the precise mechanism by which CXCL12 interacts with its receptor(s) to promote stage-specific aspects of HSC generation.

Altogether, our studies identified molecular interactions predicted by single-cell transcriptomics to establish unique engineered conditions sufficient to support the generation of engrafting HSCs from embryonic hemogenic precursors. Building on this genome-wide map of the “interactome” regulating HSC development in the AGM vascular niche, future studies will be necessary to characterize the role of additional niche factors identified by our ligand–receptor analysis in further optimizing HSC generation in vitro, particularly from earlier stages of development. Based on dynamic changes in the expression of genes involved in key signal pathways, such as Notch, Wnt, and TGF-β, we hypothesize that stage-specific modulation of these pathways during culture in the engineered niche will also enable further optimization of HSC specification and self-renewal. Furthermore, we hypothesize that signals derived from non-endothelial AGM niche cells, such as surrounding mesenchymal populations, may also be critical to optimally support HSC generation ex vivo. Altogether, our studies represent a significant step toward precisely defining the niche factors required for HSC generation, which will be critical for advancing therapeutic applications such as disease modeling and cellular therapies from human pluripotent stem cells.

### Limitations of the study

Although the V^+^61^+^E^+^ immunophenotype enabled enrichment of cells with HSC potential at different stages of maturation from HE to HSC in the AGM, this population remains functionally heterogeneous at the single-cell level, and in silico analysis was required to further resolve cells with HSC potential in our scRNA-seq data. Thus, although we identified cells co-expressing these markers within intra-aortic hematopoietic clusters of the AGM region by immunostaining, this approach is insufficient to localize HSC precursors with any degree of certainty in vivo. To our knowledge, there are no existing surface markers or single-gene reporters that can be used to confidently localize pre-HSC/HSC at single-cell resolution in the AGM at this stage. Future studies that leverage rapidly evolving technologies in spatially resolved single-cell multi-omics will be critical to adding more precise spatial information about how HSCs emerge from HE and the intercellular signaling interactions supporting this process in the AGM in vivo.

## Methods

### Reagents

Source and catalog numbers for key reagents and antibodies are listed in Supplementary Table 1.

### Mice

Wild-type C57Bl6/J7 (CD45.2) and congenic C57BL/6.SJL-Ly5.1-Pep3b (CD45.1) mice were bred at the Fred Hutchinson Cancer Research Center. C57Bl6/J7 CD45.2 mice were used for timed matings. Mice between 6 and 10 weeks of age used for most experiments were housed in individually ventilated and HEPA-filtered microisolator cage environments, with room temperature maintained between 21 and 24 degrees Celsius and humidity between 30% and 70%, in light cycles of 12 h on/12 h off. All animal studies were conducted in accordance with the NIH guidelines for the humane treatment of animals and were approved by the Institutional Animal Care and Use Committee at the Fred Hutchinson Cancer Research Center (Protocol #50765).

### Embryo dissections and cell sorting

Embryo AGM tissues (or P-Sp, para-aortic splanchnopleura—precursor region to the AGM prior to E10) were dissected from embryos harvested from pregnant C57Bl6/J7 (CD45.2) female mice as previously described^27^. Embryo age was precisely timed by counting somite pairs (sp), defined as follows (except where more specifically indicated in the figures): E9 (21-29sp), E10 (30-39 sp), and E11 (40-47sp). Dissected AGM/P-Sp tissues were treated with 0.25% collagenase for 25 min at 37°C, pipetted to single-cell suspension, and washed with PBS containing 10% fetal bovine serum (FBS). Cells were incubated with anti-mouse CD16/CD32 (FcRII block) and stained with monoclonal antibodies as indicated. A detailed list of all antibodies used is shown in Supplementary Table 1. For most experiments, to isolate hemogenic endothelial populations, a combination of anti-mouse VE-Cadherin PECy7, EPCR/CD201 PerCP-eFluor710, and CD61 APC were used, with or without additional anti-mouse antibodies for CD45 (PE or FITC), CD41 (PE or AF488), CD49d (FITC or PE), or CXCR4 (PE), as indicated in the results section. Relevant isotype control antibodies were used to set gates. 4′,6-diamidino-2-phenylindole (DAPI) staining was used to gate out dead cells. All reagents for cell staining were diluted at 1:200 in PBS with 10% FBS and staining was carried out on the ice or at 4°C. Cells were sorted on a BD FACSAria II equipped with BD FACSDiva Software (v9) with index sorting capability. For index-sorted single cells, sorting was performed in single-cell mode with maximum purity mask settings to minimize contaminating cells.

### AGM-EC generation

AGM-EC were derived as previously described^27^ and further detailed in a protocol available at Nature Protocol Exchange (https://protocolexchange.researchsquare.com/article/pex-986/v1). In brief, AGM dissected from pooled litters of embryos at similar embryonic stages were sorted by FACS for endothelial cells as VE-cadherin^+^CD45^−^CD41^−^. AGM-EC for experiments in Fig. 1 were pooled from late E10/early E11 embryos based on somite staging (EC1 40-44sp, EC2 38-42sp, EC3 42-46sp, EC4 40-42sp). Sorted cells were cultured on 48-well tissue culture plates (density >20,000 cells per well) pre-treated with 5 μg/ml RetroNectin (r-fibronectin CH-296), in EC media (consisting of IMDM with 20% FBS, penicillin/streptomycin, l-glutamine, heparin 0.1 mg/ml, and endothelial mitogen 100 μg/ml). For initial culture, EC media was supplemented with VEGF (50 ng/ml), CHIR009921 (5 μM), and SB431542 (10 μM). Following 1–2 days of culture, surviving cells (which form scattered, small tightly adherent colonies) were infected by lentivirus with pCCL-PGK myrAkt construct, as previously described^99^. When confluent, cells were serially split to 24-well, six-well, and T75 tissue culture plates (pre-treated with 0.1% gelatin) in EC media without added VEGF, CHIR09921, or SB431542. The first passage to T75 flask was considered passage 0, with subsequent passages every 3–4 days plated at ~5 × 10^5^ cells per T75 flask. All AGM-EC derivations were tested by plating cells at 4 × 10^4^ cells/24 wells in X-vivo 20 media to ensure that a confluent layer of viable ECs was maintained for at least 7 days in serum-free conditions. Endothelial identity/purity for each independently derived AGM-EC was confirmed by FACS for surface expression of VE-Cadherin. Multiple, independently derived AGM-EC were frozen at low passage at 1 × 10^6^ cells/vial for subsequent experiments. For co-culture experiments to compare HSC-supportive capacity, AGM-EC from multiple derivations were thawed in parallel from low passage aliquots onto gelatin-treated T75 flasks in EC media above, passaged every 3–4 days when confluent, and used at similar passage number for serum-free co-culture assays with freshly sorted hemogenic precursors, as described below. For single cell-RNA-sequencing (scRNA-seq) experiments, AGM-EC from multiple derivations were thawed in parallel from low passage aliquots onto gelatin-treated T75 flasks in EC media. Confluent EC was cultured in serum-free medial (X-vivo 20) 24 h prior to harvest by treatment with TrypLE Express, then resuspended in PBS/10% FBS at 4°C, washed twice in PBS in 0.04% ultrapure BSA, and resuspended PBS with 0.04% ultrapure BSA in on ice for scRNA-seq, as described below.

### AGM-EC co-culture

For co-culture experiments, AGM-EC at passage 15 or less were plated 24–48 h prior to initiation of co-culture at a density of 1 × 10^4^ cells per well to gelatin-treated 96-well tissue culture plates for single-cell index co-culture or 4 × 10^4^ cells per 24-well for bulk co-culture. For single-cell index co-culture, AGM-derived hemogenic cells were individually index sorted to each well of 96-well containing AGM-EC in serum-free media consisting of X-vivo 20 with recombinant cytokines: SCF at 100 ng/ml, and IL-3 and TPO each at 20 ng/ml. Following 5–7 days of co-culture, each well was visualized for hematopoietic colony formation and 50% of cells were harvested by vigorous pipetting for phenotypic analysis by flow cytometry, and in some experiments, remaining cells were used for confirmatory transplantation assay as previously described^28,29^. For co-culture of bulk populations, sorted cells were resuspended in serum-free culture media with cytokines, as above, and plated at 1–2 embryo equivalent of cells per 24-well containing AGM-EC. Following 6–7 days of co-culture, hematopoietic progeny were harvested by vigorous pipetting for subsequent analysis by flow cytometry and transplantation assays, as described below.

### Engineered culture conditions

Delta1ext-IgG (Dll1-Fc) was generated as previously described^100^. In all, 48-well non-tissue culture-treated plates (Falcon; BD Biosciences) were incubated with PBS containing: Dll1-Fc (2.5 μg/ml), anti-Notch1 (clone HMN1-12), and anti-Notch2 (clone HMN2-35) antibodies (each at 10 μg/ml), or Armenian Hamster IgG Isotype Control Antibody, together with 5 μg/ml recombinant fibronectin fragment (FN-CH-296), recombinant mouse VCAM1/CD106 Fc Chimera, or human IgG control antibody, as indicated in the Results section. Following overnight incubation at 4 °C, wells were washed twice with excess PBS prior to adding media. Serum-free media consisted of StemSpan SFEM II with recombinant cytokines: SCF at 100 ng/ml, and IL-3 and TPO each at 20 ng/ml, and small-molecule inhibitor of TGFBR (10 μM LY364947; from stock 10 mM solution in DMSO). For some experiments, as indicated, cells were cultured in the above media with or without recombinant murine CXCL12 (100 ng/ml). Freshly sorted VE-cadherin^+^ECPR^+^CD61^+^ cells from dissected E9, E10 or E11 AGM (as indicated) were resuspended in media above and added to coated 48-well plates (~1–2 embryo equivalent of cells per 48-well) in a total volume of 500 μl/48-well. Cells were harvested for phenotypic analysis by flow cytometry and transplantation assays at days 6–7 of culture, as described below. A more detailed protocol for engineered niche co-culture is also available at Nature Protocol Exchange (https://protocolexchange.researchsquare.com/).

### Flow cytometry analysis of cultured cells

Following co-culture, a fraction of the generated hematopoietic progeny was harvested by vigorous pipetting from the EC layer or engineered conditions for analysis of surface phenotype by flow cytometry (~10% per well of cells cultured in bulk on AGM-EC or engineered conditions, or 50% of cells generated following single-cell index culture on AGM-EC). Cells were spun and resuspended in PBS with 2% FBS, pre-incubated with anti-mouse CD16/CD32 (FcRII block), and then stained with the following anti-mouse monoclonal antibodies: VE-Cadherin-PeCy7, CD45-PerCP, Gr1-FITC, F4/80-FITC, Sca1-APC, and EPCR-PE, each at 1:200 dilution. DAPI was used to exclude dead cells. Flow cytometry was performed on a Becton Dickinson Canto 2 and data were analyzed using FlowJo Software (version 10.1). Cells with HSC potential were identified phenotypically as VE-cadherin^-/low^CD45^+^Gr1^−^F4/80^−^Sca1^high^EPCR^high^. We previously showed that detection of cells with this HSC phenotype following in vitro culture correlated with long-term multilineage engraftment as measured by transplantation assays performed in parallel, whereas hematopoietic progeny without detectable phenotypic HSC did not provide detectable long-term multilineage hematopoietic engraftment^28,29^.

### Transplantation assays

Following co-culture, a fraction of the generated hematopoietic progeny was harvested by vigorous pipetting from the EC layer or engineered conditions, washed with PBS with 2% FBS, and resuspended in 100 μl PBS/2% FBS per mouse transplanted. For bulk co-culture experiments on AGM-EC or engineered conditions, the remaining 90% of cells in each well following flow cytometry analysis were pooled, washed with PBS with 2% FBS, and resuspended at 1–2 embryo equivalent of cells per 100 μl PBS/2% FBS, and combined with 5 × 10^4^ whole marrow cells from adult congenic C57BL/6.SJL-Ly5.1-Pep3b (CD45.1) mice in 100 μl PBS/2% FBS to provide hematopoietic rescue. For some single-cell index assays, to validate the presence of functional HSC in colonies containing hematopoietic progeny with phenotypic HSC (VE-cadherin^−/low^CD45^+^Gr1^−^F4/80^−^Sca1^high^EPCR^high^) following co-culture, the remaining 50% volume of each 96-well was harvested for transplantation to individual mice, resuspended in 100 μl, and combined with 5 × 10^4^ CD45.1 whole marrow cells in 100 μl PBS/2% FBS. Cell suspensions (200 μl total volume/mouse) were injected into lethally irradiated (1000 cGy using a Cesium source) congenic CD45.1 adult recipients via the tail vein. For some experiments, secondary transplants were performed by transplanting 2 × 10^6^ whole bone marrow cells harvested from primary recipients to lethally irradiated CD45.1 secondary recipients via the tail vein. Flow cytometry analysis of peripheral blood obtained by retro-orbital bleeds was performed at 16 to 24 weeks following transplantation. Lineage-specific staining for the donor (CD45.2) and recipient/rescue (CD45.1) cells from peripheral blood was performed as previously described^27^, using anti-mouse monoclonal antibodies indicated in Supplementary Table 1: CD45.1, CD45.2, CD3, CD19, Gr1, and F4/80. Multilineage engraftment was defined as >5% donor (CD45.2) contribution to the peripheral blood with the contribution to each lineage of donor myeloid (Gr1 and F4/80), B cells (CD19), and T cells (CD3) detected at ≥0.5% at 16–24 weeks post transplant, as indicated.

### Quantification of HSC and non-HSC colony-forming cells

Following single-cell index co-cultures described above, each individual sorted cell was classified based on its HSC potential. Specifically, single cells giving rise to colonies with detectable HSC by phenotype and/or transplantation analysis were categorized as HSC colony-forming cells (HSC CFC); those giving rise to colonies of CD45^+^ hematopoietic progeny lacking phenotypic or functional HSC were categorized as non-HSC colony-forming cells (non-HSC CFC), and those failing to give rise to detectable hematopoietic colonies were indicated as “no colony.” For each embryonic stage (E9-E10, E10-E11, and E11), results from multiple, pooled index sort experiments were combined to quantify the percentage of detectable HSC CFC and non-HSC CFC within the VE-Cadherin^+^CD61^+^EPCR^+^ (V^+^61^+^E^+^) sort gate and the total number of HSC CFC detected per AGM (calculated based on the number of HSC CFC detected, the number of embryo equivalents used, and the fraction of cells in FACS that were index sorted, for each experiment). (Summarized results in Supplementary Fig. 3e). The predicted range in numbers of HSC precursors in the scRNA-seq data obtained from E10 and E11 AGM-derived V^+^61^+^E^+^ cells (in Fig. 3h) was estimated using the range of HSC CFC detected in this compiled data at equivalent stages.

### Fluorescent multiplex Immunohistochemistry (IHC)

E10.5 embryos (38–39 sp) were dissected from extraembryonic tissues and the rostral portion was removed (heart and anterior). Embryos were fixed for ~3 days (76 h) in 10% neutral-buffered formalin at room temperature, transferred to 70% ethanol, and processed for paraffin embedding. Formalin-fixed paraffin-embedded tissues were sectioned at four microns throughout the entire embryo in transverse sections taken roughly perpendicular to the long (caudal-rostral) axis of the embryo, transferred onto positively charged slides, and baked for 30 min at 65 °C. H&E staining was performed on alternating slides to facilitate the identification of embryonic structures, including hematopoietic clusters within the dorsal aorta of the AGM region. Unstained slides selected for IHC were deparaffinized and stained on a Leica BOND RX stainer (Leica, Buffalo Grove, IL) using Leica Bond reagents for dewaxing (Dewax Solution), antigen retrieval/antibody stripping (Epitope Retrieval Solution 2), and rinsing after each step (Bond Wash Solution). Antigen retrieval and antibody stripping steps were performed at 100 °C, with all other steps at ambient temperature. Endogenous peroxidase was blocked with 3% H_2_O_2_ for 8 min followed by protein blocking with 10% normal mouse immune serum diluted in TCT buffer (0.05 M Tris, 0.15 M NaCl, 0.25% casein, 0.1% Tween 20, pH 7.6) for 30 min. The first primary antibody (position 1, Table 1) was applied for 60 min followed by the secondary antibody application for 10 min and the application of the tertiary TSA-amplification reagent (PerkinElmer OPAL fluor) for 10 min. A high stringency wash was performed after the secondary and tertiary applications using high-salt TBST solution (0.05 M Tris, 0.3 M NaCl, and 0.1% Tween 20, pH 7.2–7.6). Species-specific HRP polymer was used for all secondary applications, either Leica’s PowerVision Poly-HRP Anti-Rabbit Detection, or the ImmPress Anti-Goat IgG Polymer Detection Kit (Vector Labs, Burlingame, CA). The primary and secondary antibodies were stripped with retrieval solution for 20 min before repeating the process with the second primary antibody (position 2, Table 1) starting with a new application of 3% H_2_O_2_. The process was repeated until all three positions were completed (Table 1); however, there was no stripping step after the final position. Slides were removed from the stainer and stained with DAPI for 5 min, rinsed for 5 min, and coverslipped with Prolong Gold Antifade reagent (Invitrogen/Life Technologies, Grand Island, NY). Slides were cured at room temperature, then whole slide images were acquired on an Aperio FL fluorescent slide scanner (Leica Biosystems, Buffalo Grove, IL).

**Table 1:** Antibodies used for fluorescent multiplex IHC.

Position	Antibody	Clone/ Host	Manufacturer/catalog number	Dilution/concentration	Secondary	Opal fye
1	EPCR	Polyclonal; Rabbit	Invitrogen PA5-81459	Stock: 1 mg/ml 1:8000	Powervision rabbit HRP	Opal 650
2	CD61	SJ19-09; Rabbit	Novus NBP2-67416	Stock: 1 mg/ml 1:6000	Powervision rabbit HRP	Opal 570
3	VE-cadherin	Polyclonal; goat	R&D Sys AF1002	Stock: 200 µg/ml 1:500	ImmPRESS goat HRP	Opal 520

### Single-cell RNA sequencing

For single-cell RNA-sequencing (scRNA-seq) studies, freshly sorted AGM-derived cells or cells harvested following culture (as indicated above) were washed with PBS containing 0.04% ultrapure BSA and resuspended in 0.04% ultrapure BSA in PBS on ice. Cell suspensions were loaded into the Chromium Single Cell B Chip (10X Genomics) and processed in the Chromium single cell controller (10X Genomics), targeting 3500 cells per lane from freshly sorted AGM-derived cells, cultured AGM-EC, or progeny of AGM-derived cells following culture on AGM-EC or engineered conditions, or the remaining 50% progeny of clonal AGM-derived V^+^E^+^61^+^ cells following AGM-EC co-culture and flow cytometry analysis (estimated to contain <500 cells). The 10X Genomics Version 2 single cell 3’ kit was used to prepare single-cell mRNA libraries with the Chromium i7 Multiplex Kit, according to manufacturer protocols. Sequencing was performed for pooled libraries from each sample on an Illumina NextSeq 500 using the 75 cycle, high output kit, targeting a minimum of 50,000 reads per cell. For AGM-EC, scRNA-seq was performed from individual samples from each AGM-EC line, resulting in the acquisition of >1000 individual high-quality cell sequences per cell line. For AGM-derived V^+^61^+^E^+^ cells, scRNA-seq was performed in two independent experiments from cells sorted from embryos pooled at E10 (46 embryos, 31–39 sp) and E11 (54 embryos, 40–45sp), resulting in acquisition of >3000 individual high-quality cell sequences between E10 and E11. For clonal progeny of AGM-derived V^+^61^+^E^+^ cells (HSC CFC) generated following AGM-EC co-culture, scRNA-seq was performed from two independent, representative colonies, resulting in the acquisition of 152 high-quality cell sequences from one colony and 991 high-quality cell sequences from the second colony. For the progeny of AGM-derived V^+^61^+^E^+^ cells following culture on AGM-EC or engineered conditions, scRNA-seq was performed from cells freshly harvested from each condition, processed and sequenced in parallel to minimize batch effects, resulting in acquisition of 6887 high-quality cell sequences (from AGM-EC co-culture conditions) and 4871 high-quality cell sequences (from engineered conditions). All sequencing data have been uploaded to NCBI GEO (accession number GSE145886).

### Single-cell transcriptome analysis and quality control

The Cell Ranger 2.1.1 pipeline (10X Genomics) was used to align reads to the mm10 reference genome and generate a feature barcode matrix, filtering low-quality cells using default parameters. The Monocle (v.2.99.3) platform was used for downstream analysis (in R v.3.6.1), combining data for cells from each individual sample and/or replicate (as described above) for downstream analysis, using a negative binomial model of distribution with fixed variance, normalizing expression matrices by size factors. Counts for UMI (unique molecular identifiers) and unique genes expressed per cell are shown in boxplots in supplementary figures for each sample (showing median values and interquartile ranges; upper/lower whiskers show 1.5× interquartile range with outliers shown as individual dots).

### Dimensionality reduction and cluster analysis

Genes used for clustering were selected based on dispersion thresholds, and the preprocessCDS() function was used to project the data onto the top principal components (excluding principal components that contributed little to the overall variance). UMAP was used for dimensionality reduction with the reduceDimension function, with reduction_method = ‘UMAP’. Clustering was performed by Louvain method with the clusterCells function.

### Differential gene expression between clusters and gene ontology analysis

Differential gene expression between clusters (for Fig. 1) was performed using spatial correlation analysis with the principalGraphTest and find_cluster_markers functions, using a Moran’s I threshold of 0.25, filtering genes based on specificity >0.75. Gene ontology analysis was performed on genes identified as differentially expressed between clusters by PANTHER overrepresentation test with default settings (Fisher’s Exact, FDR correction) using annotation data sets for GO biological process and GO molecular function (Release version 2019-07-03, http://geneontology.org/).

### Gene-set scores

Gene-set scores (for Fig. 1, supplementary Fig. 9) were calculated for every single cell as the log-transformed sum of the size factor-normalized expression for each gene within a given GO term as indicated (see Supplementary Data 2). Gene-set scores (for Fig. 3, Supplementary Fig. 6, 7, 8h) were calculated for every single cell as the log-transformed sum of the size factor-normalized expression for each gene in published signature/marker gene sets identified by scRNA-seq analysis, including: arterial EC marker genes^50^, “HSC-primed HE”^26^, pre-HSC (type I and type II) isolated from E11 AGM (using the subset of 34 genes also expressed in HSC)^22^, functional HSC isolated from E11 AGM^30^, and adult marrow-derived HSC isolated by index sorting (29 genes representing a molecular overlap signature of HSC isolated by different sorting strategies)^31^ (see Supplementary Data 6). Violin plots of gene-set scores for each cell type were generated using ggplot function with geom_violin() and geom_boxplot() (boxplots show median values and interquartile ranges; upper/lower whiskers show 1.5X interquartile range) in the ggplot2 package (v 3.3.2).

### Cell type classification

Cell type classification was performed using the classifyCells function (method set to “markers-only”), with marker gene sets based on established cell type-specific genes, as indicated (marker genes listed in Supplementary Fig. 5f, g, Supplementary Fig. 8e, Supplementary Fig. 11e). Only cells identified by strict expression of indicated genes are classified as a given cell type by this method. This approach was chosen to maximize cell type specificity for downstream analysis of expressed receptors and ligands, however, leaving a subset of cell types unclassified rather than imputing cell types for all cells in the scRNA-seq data set.

### Pseudotime analysis and differential gene expression over pseudotime

Pseudotime trajectory analysis was performed using the learnGraph function, with RGE_method set to SimplePPT. To identify genes that vary over pseudotime representing the EC/HE to hematopoietic transition from primary AGM-derived V^+^61^+^E^+^ cells in vivo (Fig. 3d, e, Supplementary Data 3) and over pseudotime representing HSC to HPC differentiation in vitro (Fig. 4g, Supplementary Data 4), differential gene expression was performed using the differentialGeneTest function, with fullModelFormulaStr set to Pseudotime, limited to genes with detected expression in >5% of cells and a significance cutoff of *q* value < 0.01.

### Ligand–receptor expression analysis

To identify genes coding for ligands expressed in niche endothelial cells, genes were first limited to those with detectable UMI in at least 10% of cells (threshold chosen to account for transcript drop-out inherent in scRNA-seq data and potential heterogeneity in endothelial cells) in 1) HSC-supportive AGM-EC #1, #2, and #3, (cluster 1, from Figs. 1 or 2) primary AGM-derived cells identified by cell type classification as “arterial EC” (from Fig. 3c–g). These genes were then cross referenced with all potential ligands in the ligand–receptor database published by Ramilowski et al.^67^, using mouse gene names corresponding to human gene names in the published database (comprehensive list of ligands identified is provided in Supplementary Data 5). To identify genes coding for receptors expressed in primary AGM-derived cells identified by cell type classification as “HSC precursor” type (From Fig. 3h), genes were first limited to those with detectable UMI in 5% of cells of this type (threshold was chosen to account for transcript drop-out inherent in scRNA-seq data as well as expected heterogeneity and asynchrony in this population representing HSC precursors transitioning from HE to pre-HSC/HSC, so as not to exclude genes expressed at low level or in rare transitional states). These genes were then cross referenced with all potential receptors in the ligand–receptor database published by Ramilowski et al.^67^ (comprehensive list of receptors expressed in HSC precursors is provided in Supplementary Data 5). To identify genes coding for receptors expressed in HSC generated in vitro (i.e., in the progeny of clonal AGM-derived V^+^61^+^E^+^ cells following AGM-EC co-culture in Fig. 4), genes were first limited to those with detectable UMI in at least 20% of cells of “HSC” type (threshold was chosen to account for transcript drop-out inherent in scRNA-seq data as well and potential heterogeneity in HSCs generated in vitro, such as based on cell cycle status). These genes were then cross referenced with all potential receptors in the ligand–receptor database published by Ramilowski et al.^67^ (comprehensive list of receptors expressed in HSCs is provided in Supplementary Data 5). All potential ligand–receptor pairings from this database were then determined for interactions between AGM-EC (using ligand data from primary AGM-derived arterial EC or HSC-supportive AGM-EC stroma) and primary AGM-derived HSC precursors or in vitro generated HSC (Fig. 5 and Supplementary Data 5). Circos plots for visualization of ligand–receptor interactions for selected signal pathways (Fig. 5i–viii) were generated using the Circlize package (v0.4.8)^101^, modified from the iTALK package (v0.1.0)^102^. Ligands were categorized as those detected in either primary AGM-derived arterial EC, HSC-supportive AGM-EC lines, or both; or as those representing cytokines added exogenously during AGM-EC co-culture (i.e., IL-3, SCF, TPO) (Fig. 5 legend, left panel). Receptors were categorized as those detected as described above in primary AGM-derived cells identified by cell type classification as “HSC precursor” type or following AGM-EC co-culture identified by cell type classification as “HSC” type (Fig. 5 legend, right panel). Receptors that were detected to significantly vary over pseudotime analysis of HSC precursors (as described above, see Fig. 3d-e, Supplementary Data 3) were also categorized as enriched in early pseudotime (i.e., expression enriched in HE greater than pre-HSC) or late pseudotime (i.e., expression enriched in pre-HSC greater that HE) (Fig. 5. Legend, right panel). Receptors that were detected to significantly vary over pseudotime in the analysis of HSC differentiation to HPC in vitro (as described above, see Fig. 4h, Supplementary Data 4) were also categorized as enriched in early pseudotime (i.e., expression enriched in HSC greater than HPC) or not significantly changed over pseudotime (Fig. 5. Legend, right panel). Ligand–receptor interactions involving ligands that were significantly enriched in expression in HSC-supportive versus non-supportive AGM-EC (from Supplementary Data 1) are indicated (Fig. 5 legend, center panel, yellow arrows).

### Statistics and reproducibility

For statistical analysis between groups, two-sided, unpaired Student’s *t* test or two-sided, unpaired Wilcoxon Rank Sum Test (for gene-set scores, ggupbr package v0.4.0) was used to calculate *P* values where indicated. For each figure, unless otherwise indicated, results were replicated in at least 3 independent experiments.

### Reporting summary

Further information on research design is available in the Nature Research Reporting Summary linked to this article.

## Supplementary information

Supplementary Information

Description of Additional Supplementary Files

Supplementary Data 1

Supplementary Data 2

Supplementary Data 3

Supplementary Data 4

Supplementary Data 5

Supplementary Data 6

Reporting Summary

## Data Availability

The sequencing data generated in this study have been deposited in the NCBI Gene Expression Omnibus (GEO) database under accession code GSE145886. All raw and processed sequencing data (fastq files, CellRanger output files, Monocle cell data sets) necessary to interpret, verify, and extend the research in the article are publicly available with no restrictions on access. Source data are provided with this paper.

## Source data

Source Data

## Code availability

R scripts used for the analysis of scRNA-seq data have been deposited at Github and are publicly available as of the date of publication. Github repository: https://github.com/FredHutch/hadland-etal-2022; Zenodo: https://zenodo.org/record/5806809#.YcphyyyIb0o.

## References

[1] Hadland B, Yoshimoto M (2018). Many layers of embryonic hematopoiesis: new insights into B-cell ontogeny and the origin of hematopoietic stem cells. Exp. Hematol..

[2] Gordon-Keylock S, Sobiesiak M, Rybtsov S, Moore K, Medvinsky A (2013). Mouse extraembryonic arterial vessels harbor precursors capable of maturing into definitive HSCs. Blood.

[3] Medvinsky A, Dzierzak E (1996). Definitive hematopoiesis is autonomously initiated by the AGM region. Cell.

[4] Müller AM, Medvinsky A, Strouboulis J, Grosveld F, Dzierzak E (1994). Development of hematopoietic stem cell activity in the mouse embryo. Immunity.

[5] Hadland BK (2004). A requirement for Notch1 distinguishes 2 phases of definitive hematopoiesis during development. Blood.

[6] Gama-Norton L (2015). Notch signal strength controls cell fate in the haemogenic endothelium. Nat. Commun..

[7] Clarke RL (2013). The expression of Sox17 identifies and regulates haemogenic endothelium. Nat. Cell Biol..

[8] Uenishi GI (2018). NOTCH signaling specifies arterial-type definitive hemogenic endothelium from human pluripotent stem cells. Nat. Commun..

[9] Dou DR (2016). Medial HOXA genes demarcate haematopoietic stem cell fate during human development. Nat. Cell Biol..

[10] Bonkhofer F (2019). Blood stem cell-forming haemogenic endothelium in zebrafish derives from arterial endothelium. Nat. Commun..

[11] Ng ES (2016). Differentiation of human embryonic stem cells to HOXA. Nat. Biotechnol..

[12] Gao L (2018). RUNX1 and the endothelial origin of blood. Exp. Hematol..

[13] Kumaravelu P (2002). Quantitative developmental anatomy of definitive haematopoietic stem cells/long-term repopulating units (HSC/RUs): role of the aorta-gonad-mesonephros (AGM) region and the yolk sac in colonisation of the mouse embryonic liver. Development.

[14] Rybtsov S, Ivanovs A, Zhao S, Medvinsky A (2016). Concealed expansion of immature precursors underpins acute burst of adult HSC activity in foetal liver. Development.

[15] Yoder MC, Hiatt K, Mukherjee P (1997). In vivo repopulating hematopoietic stem cells are present in the murine yolk sac at day 9.0 postcoitus. Proc. Natl. Acad. Sci. USA.

[16] Rybtsov S (2014). Tracing the origin of the HSC hierarchy reveals an SCF-dependent, IL-3-independent CD43(-) embryonic precursor. Stem Cell Rep..

[17] Rybtsov S (2011). Hierarchical organization and early hematopoietic specification of the developing HSC lineage in the AGM region. J. Exp. Med..

[18] Taoudi S (2005). Progressive divergence of definitive haematopoietic stem cells from the endothelial compartment does not depend on contact with the foetal liver. Development.

[19] Yokomizo T, Dzierzak E (2010). Three-dimensional cartography of hematopoietic clusters in the vasculature of whole mouse embryos. Development.

[20] Pijuan-Sala B, Guibentif C, Göttgens B (2018). Single-cell transcriptional profiling: a window into embryonic cell-type specification. Nat. Rev. Mol. Cell Biol..

[21] Zhu, Q. et al. Developmental trajectory of pre-hematopoietic stem cell formation from endothelium. *Blood* **136**, 845–856 (2020). 10.1182/blood.2020004801 PMC7426642 32392346

[22] Zhou F (2016). Tracing haematopoietic stem cell formation at single-cell resolution. Nature.

[23] Baron CS (2018). Single-cell transcriptomics reveal the dynamic of haematopoietic stem cell production in the aorta. Nat. Commun..

[24] Oatley M (2020). Single-cell transcriptomics identifies CD44 as a marker and regulator of endothelial to haematopoietic transition. Nat. Commun..

[25] Zeng Y (2019). Tracing the first hematopoietic stem cell generation in human embryo by single-cell RNA sequencing. Cell Res..

[26] Hou S (2020). Embryonic endothelial evolution towards first hematopoietic stem cells revealed by single-cell transcriptomic and functional analyses. Cell Res..

[27] Hadland BK (2015). Endothelium and NOTCH specify and amplify aorta-gonad-mesonephros-derived hematopoietic stem cells. J. Clin. Invest..

[28] Hadland BK (2017). A common origin for B-1a and B-2 lymphocytes in clonal pre- hematopoietic stem cells. Stem Cell Rep..

[29] Hadland, B. K., Varnum-Finney, B., Nourigat-Mckay, C., Flowers, D. & Bernstein, I. D. Clonal analysis of embryonic hematopoietic stem cell precursors using single cell index sorting combined with endothelial cell niche co-culture. *J. Vis. Exp.* **8**, 56973 (2018). 10.3791/56973 PMC6101165 29806841

[30] Vink CS (2020). Iterative single-cell analyses define the transcriptome of the first functional hematopoietic stem cells. Cell Rep..

[31] Wilson NK (2015). Combined single-cell functional and gene expression analysis resolves heterogeneity within stem cell populations. Cell Stem Cell.

[32] Chiang IK (2017). SoxF factors induce Notch1 expression via direct transcriptional regulation during early arterial development. Development.

[33] Corada M (2013). Sox17 is indispensable for acquisition and maintenance of arterial identity. Nat. Commun..

[34] Wilkinson RN (2009). Hedgehog and Bmp polarize hematopoietic stem cell emergence in the zebrafish dorsal aorta. Dev. Cell.

[35] Balazs AB, Fabian AJ, Esmon CT, Mulligan RC (2006). Endothelial protein C receptor (CD201) explicitly identifies hematopoietic stem cells in murine bone marrow. Blood.

[36] Iwasaki H, Arai F, Kubota Y, Dahl M, Suda T (2010). Endothelial protein C receptor-expressing hematopoietic stem cells reside in the perisinusoidal niche in fetal liver. Blood.

[37] Huang K (2016). Generation and analysis of GATA2(w/eGFP) human ESCs reveal ITGB3/CD61 as a reliable marker for defining hemogenic endothelial cells during hematopoiesis. Stem Cell Rep..

[38] Boisset JC, Clapes T, Van Der Linden R, Dzierzak E, Robin C (2013). Integrin alphaIIb (CD41) plays a role in the maintenance of hematopoietic stem cell activity in the mouse embryonic aorta. Biol. Open.

[39] Li W, Ferkowicz MJ, Johnson SA, Shelley WC, Yoder MC (2005). Endothelial cells in the early murine yolk sac give rise to CD41-expressing hematopoietic cells. Stem Cells Dev..

[40] Mikkola HK, Fujiwara Y, Schlaeger TM, Traver D, Orkin SH (2003). Expression of CD41 marks the initiation of definitive hematopoiesis in the mouse embryo. Blood.

[41] Fischer A, Schumacher N, Maier M, Sendtner M, Gessler M (2004). The Notch target genes Hey1 and Hey2 are required for embryonic vascular development. Genes Dev..

[42] Taoudi S (2008). Extensive hematopoietic stem cell generation in the AGM region via maturation of VE-cadherin+CD45+ pre-definitive HSCs. Cell Stem Cell.

[43] Qiu, X. et al. Reversed graph embedding resolves complex single-cell trajectories. *Nat. Methods* https://www.biorxiv.org/content/10.1101/110668v1 (2017). 10.1038/nmeth.4402 PMC5764547 28825705

[44] Taoudi S (2011). ERG dependence distinguishes developmental control of hematopoietic stem cell maintenance from hematopoietic specification. Genes Dev..

[45] Calvanese V (2019). MLLT3 governs human haematopoietic stem-cell self-renewal and engraftment. Nature.

[46] Mallaney C (2019). Kdm6b regulates context-dependent hematopoietic stem cell self-renewal and leukemogenesis. Leukemia.

[47] Kataoka K (2011). Evi1 is essential for hematopoietic stem cell self-renewal, and its expression marks hematopoietic cells with long-term multilineage repopulating activity. J. Exp. Med..

[48] Ficara F, Murphy MJ, Lin M, Cleary ML (2008). Pbx1 regulates self-renewal of long-term hematopoietic stem cells by maintaining their quiescence. Cell Stem Cell.

[49] Wang H (2016). SIRT6 controls hematopoietic stem cell homeostasis through epigenetic regulation of Wnt signaling. Cell Stem Cell.

[50] Kalucka J (2020). Single-cell transcriptome atlas of murine endothelial cells. Cell.

[51] Matsumoto A (2011). p57 is required for quiescence and maintenance of adult hematopoietic stem cells. Cell Stem Cell.

[52] Sawai CM (2016). Hematopoietic stem cells are the major source of multilineage hematopoiesis in adult animals. Immunity.

[53] Carrelha J (2018). Hierarchically related lineage-restricted fates of multipotent haematopoietic stem cells. Nature.

[54] Sanjuan-Pla A (2013). Platelet-biased stem cells reside at the apex of the haematopoietic stem-cell hierarchy. Nature.

[55] Acar M (2015). Deep imaging of bone marrow shows non-dividing stem cells are mainly perisinusoidal. Nature.

[56] Guiu J (2014). Identification of Cdca7 as a novel Notch transcriptional target involved in hematopoietic stem cell emergence. J. Exp. Med..

[57] Robert-Moreno A, Espinosa L, de la Pompa JL, Bigas A (2005). RBPjkappa-dependent Notch function regulates Gata2 and is essential for the formation of intra-embryonic hematopoietic cells. Development.

[58] de Pater E (2013). Gata2 is required for HSC generation and survival. J. Exp. Med..

[59] Guiu J (2013). Hes repressors are essential regulators of hematopoietic stem cell development downstream of Notch signaling. J. Exp. Med..

[60] Lizama CO (2015). Repression of arterial genes in hemogenic endothelium is sufficient for haematopoietic fate acquisition. Nat. Commun..

[61] Zhang P (2015). G protein-coupled receptor 183 facilitates endothelial-to-hematopoietic transition via Notch1 inhibition. Cell Res..

[62] Souilhol C (2016). Developing HSCs become Notch independent by the end of maturation in the AGM region. Blood.

[63] Gazit R (2014). Fgd5 identifies hematopoietic stem cells in the murine bone marrow. J. Exp. Med..

[64] Fathman JW (2014). Upregulation of CD11A on hematopoietic stem cells denotes the loss of long-term reconstitution potential. Stem Cell Rep..

[65] Kiel MJ, Yilmaz OH, Iwashita T, Terhorst C, Morrison SJ (2005). SLAM family receptors distinguish hematopoietic stem and progenitor cells and reveal endothelial niches for stem cells. Cell.

[66] Butler JM (2010). Endothelial cells are essential for the self-renewal and repopulation of Notch-dependent hematopoietic stem cells. Cell Stem Cell.

[67] Ramilowski JA (2015). A draft network of ligand-receptor-mediated multicellular signalling in human. Nat. Commun..

[68] Bigas A, Guiu J, Gama-Norton L (2013). Notch and Wnt signaling in the emergence of hematopoietic stem cells. Blood Cells Mol. Dis..

[69] Lengerke C (2008). BMP and Wnt specify hematopoietic fate by activation of the Cdx-Hox pathway. Cell Stem Cell.

[70] Burns CE (2009). A genetic screen in zebrafish defines a hierarchical network of pathways required for hematopoietic stem cell emergence. Blood.

[71] Peeters M (2009). Ventral embryonic tissues and Hedgehog proteins induce early AGM hematopoietic stem cell development. Development.

[72] Chen II (2016). EphrinB2 regulates the emergence of a hemogenic endothelium from the aorta. Sci. Rep..

[73] Esain V (2015). Cannabinoid receptor-2 regulates embryonic hematopoietic stem cell development via prostaglandin E2 and P-selectin activity. Stem Cells.

[74] Himburg HA (2010). Pleiotrophin regulates the expansion and regeneration of hematopoietic stem cells. Nat. Med..

[75] Lin, M. I. *et al*. Angiopoietin-like proteins stimulate HSPC development through interaction with notch receptor signaling. *Elife* **4**, e05544 (2015). 10.7554/eLife.05544 PMC4371382 25714926

[76] Bruns I (2014). Megakaryocytes regulate hematopoietic stem cell quiescence through CXCL4 secretion. Nat. Med..

[77] Nguyen PD (2014). Haematopoietic stem cell induction by somite-derived endothelial cells controlled by meox1. Nature.

[78] Thomas DD (2016). Insulin-like growth factor 2 modulates murine hematopoietic stem cell maintenance through upregulation of p57. Exp. Hematol..

[79] Zhang CC, Lodish HF (2004). Insulin-like growth factor 2 expressed in a novel fetal liver cell population is a growth factor for hematopoietic stem cells. Blood.

[80] Crosse EI (2020). Multi-layered spatial transcriptomics identify secretory factors promoting human hematopoietic stem cell development. Cell Stem Cell.

[81] Yvernogeau L (2020). Multispecies RNA tomography reveals regulators of hematopoietic stem cell birth in the embryonic aorta. Blood.

[82] Kim AD (2014). Discrete Notch signaling requirements in the specification of hematopoietic stem cells. EMBO J..

[83] Varnum-Finney B (2011). Notch2 governs the rate of generation of mouse long- and short-term repopulating stem cells. J. Clin. Invest.

[84] Yang LT (2005). Fringe glycosyltransferases differentially modulate Notch1 proteolysis induced by Delta1 and Jagged1. Mol. Biol. Cell.

[85] Akimov SS, Krylov D, Fleischman LF, Belkin AM (2000). Tissue transglutaminase is an integrin-binding adhesion coreceptor for fibronectin. J. Cell Biol..

[86] Dignum T (2021). Multipotent progenitors and hematopoietic stem cells arise independently from hemogenic endothelium in the mouse embryo. Cell Rep..

[87] Taoudi S, Medvinsky A (2007). Functional identification of the hematopoietic stem cell niche in the ventral domain of the embryonic dorsal aorta. Proc. Natl. Acad. Sci. USA.

[88] Souilhol C (2016). Inductive interactions mediated by interplay of asymmetric signalling underlie development of adult haematopoietic stem cells. Nat. Commun..

[89] Rho SS (2019). Rap1b promotes notch-signal-mediated hematopoietic stem cell development by enhancing integrin-mediated cell adhesion. Dev. Cell.

[90] Nie Y, Han YC, Zou YR (2008). CXCR4 is required for the quiescence of primitive hematopoietic cells. J. Exp. Med..

[91] Karpova D, Bonig H (2015). Concise review: CXCR4/CXCL12 signaling in immature hematopoiesis-lessons from pharmacological and genetic models. Stem Cells.

[92] Pillay LM, Mackowetzky KJ, Widen SA, Waskiewicz AJ (2016). Somite-derived retinoic acid regulates zebrafish hematopoietic stem cell formation. PLoS One.

[93] Park MA (2018). Activation of the arterial program drives development of definitive hemogenic endothelium with lymphoid potential. Cell Rep..

[94] Guo Y, Hangoc G, Bian H, Pelus LM, Broxmeyer HE (2005). SDF-1/CXCL12 enhances survival and chemotaxis of murine embryonic stem cells and production of primitive and definitive hematopoietic progenitor cells. Stem Cells.

[95] Ahmed T, Tsuji-Tamura K, Ogawa M (2016). CXCR4 signaling negatively modulates the bipotential state of hemogenic endothelial cells derived from embryonic stem cells by attenuating the endothelial potential. Stem Cells.

[96] Fujita M, Davari P, Takada YK, Takada Y (2018). Stromal cell-derived factor-1 (CXCL12) activates integrins by direct binding to an allosteric ligand-binding site (site 2) of integrins without CXCR4. Biochem. J..

[97] Peled A (2000). The chemokine SDF-1 activates the integrins LFA-1, VLA-4, and VLA-5 on immature human CD34(+) cells: role in transendothelial/stromal migration and engraftment of NOD/SCID mice. Blood.

[98] Peled A (1999). The chemokine SDF-1 stimulates integrin-mediated arrest of CD34(+) cells on vascular endothelium under shear flow. J. Clin. Invest..

[99] Kobayashi H (2010). Angiocrine factors from Akt-activated endothelial cells balance self-renewal and differentiation of haematopoietic stem cells. Nat. Cell Biol..

[100] Varnum-Finney B (2000). Immobilization of Notch ligand, Delta-1, is required for induction of notch signaling. J. Cell Sci..

[101] Gu Z, Gu L, Eils R, Schlesner M, Brors B (2014). circlize Implements and enhances circular visualization in R. Bioinformatics.

[102] Wang, Y. et al. iTalk: an R package to characterize and illustrate intercellular communication. *bioRxiv* https://www.biorxiv.org/content/10.1101/507871v1 (2019).

