# Supplementary material for: Engineering a niche supporting hematopoietic stem cell development using integrated single-cell transcriptomics
Source: Nat Commun. 2022 Mar 24;13:1584. doi: 10.1038/s41467-022-28781-z (PMC8948249; doi:10.1038/s41467-022-28781-z)
Supplement: Supplementary file 1 — Supplementary Information [file 41467_2022_28781_MOESM1_ESM.pdf]

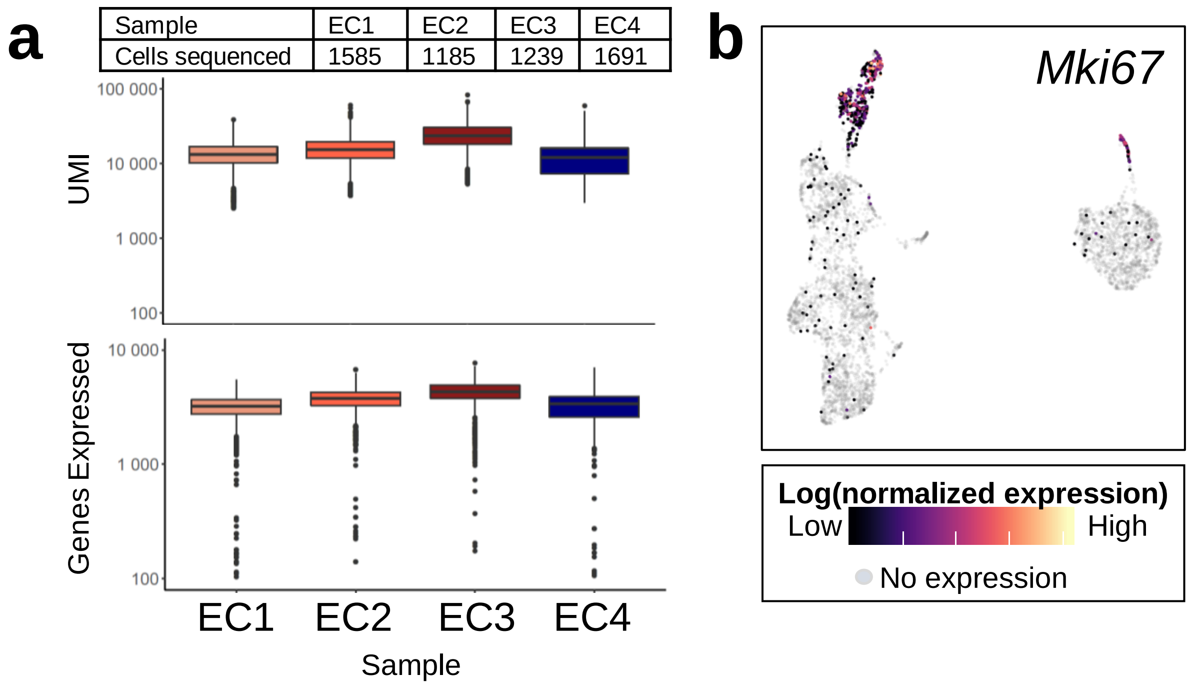

Supplementary Figure 1. **a**. Counts of UMI and unique genes expressed per cell for each AGM-EC sample (EC1-4). Boxplots show median values and interquartile ranges; upper/lower whiskers show 1.5X interquartile range with outliers shown as individual dots. **b**. Expression of cell cycle gene *Mki67,* demonstrating intra-cluster cell cycle heterogeneity. (Related to Fig. 1).

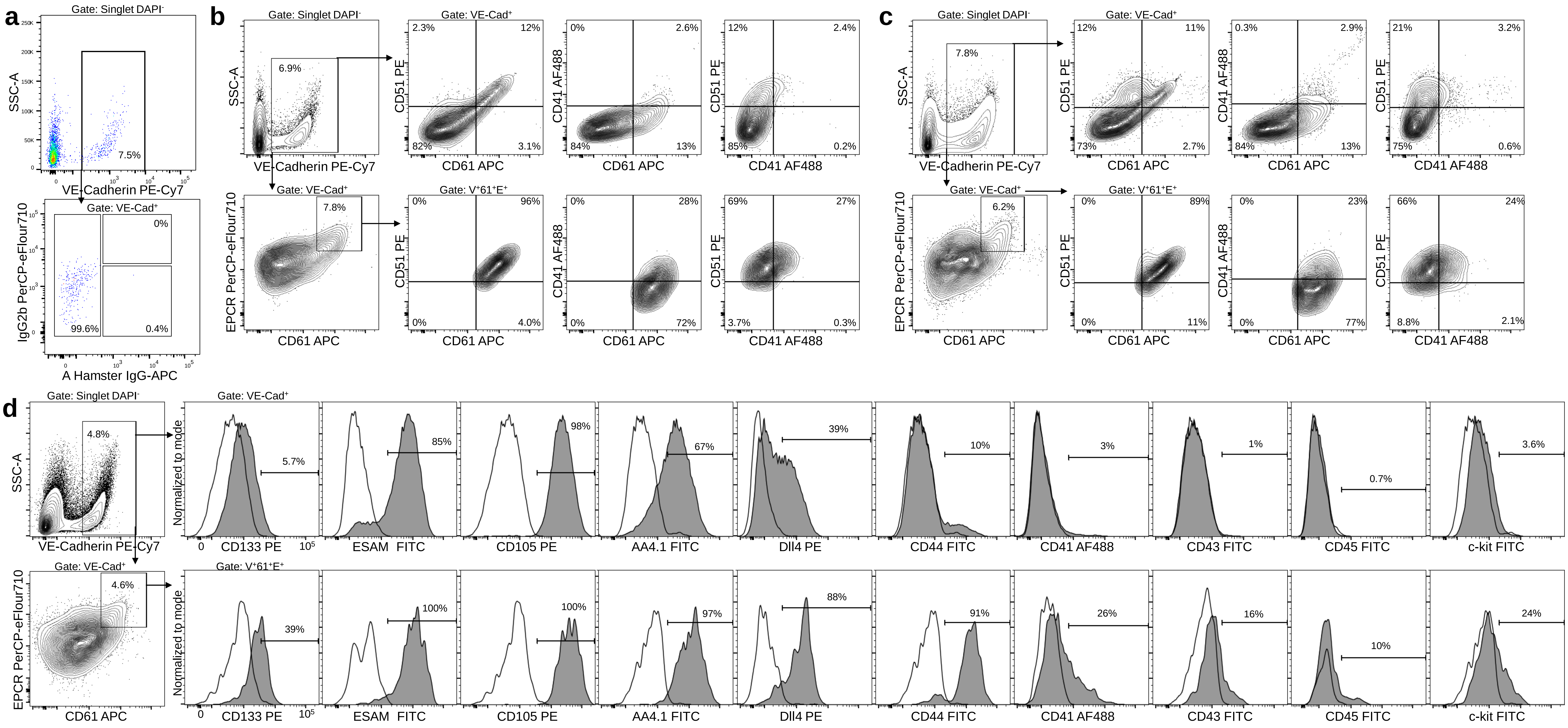

Supplementary Figure 2. a. Gating strategy with isotype controls for EPCR (IgG PerCP-eFlour710) and CD61 (IgG APC) within the population sorted as positive for VE-Cadherin. (Related to Fig. 2a). **b-c.** Expression of CD61 integrin co-receptors CD51 and CD41 in the total AGM-derived VE-cadherin+ population (upper panel) and the V+61+E+ subset (lower panel) at b) late E9 (26-28 sp) and late E10 (35-39 sp) **d.** Expression of surface markers by flow in the total AGM-derived VE-cadherin+ population (upper panel) and the V+61+E+ subset (lower panel) at E10-E11 (35-43 sp). Unshaded histogram in each panel represents staining with relevant isotype controls. (Related to Fig. 2).

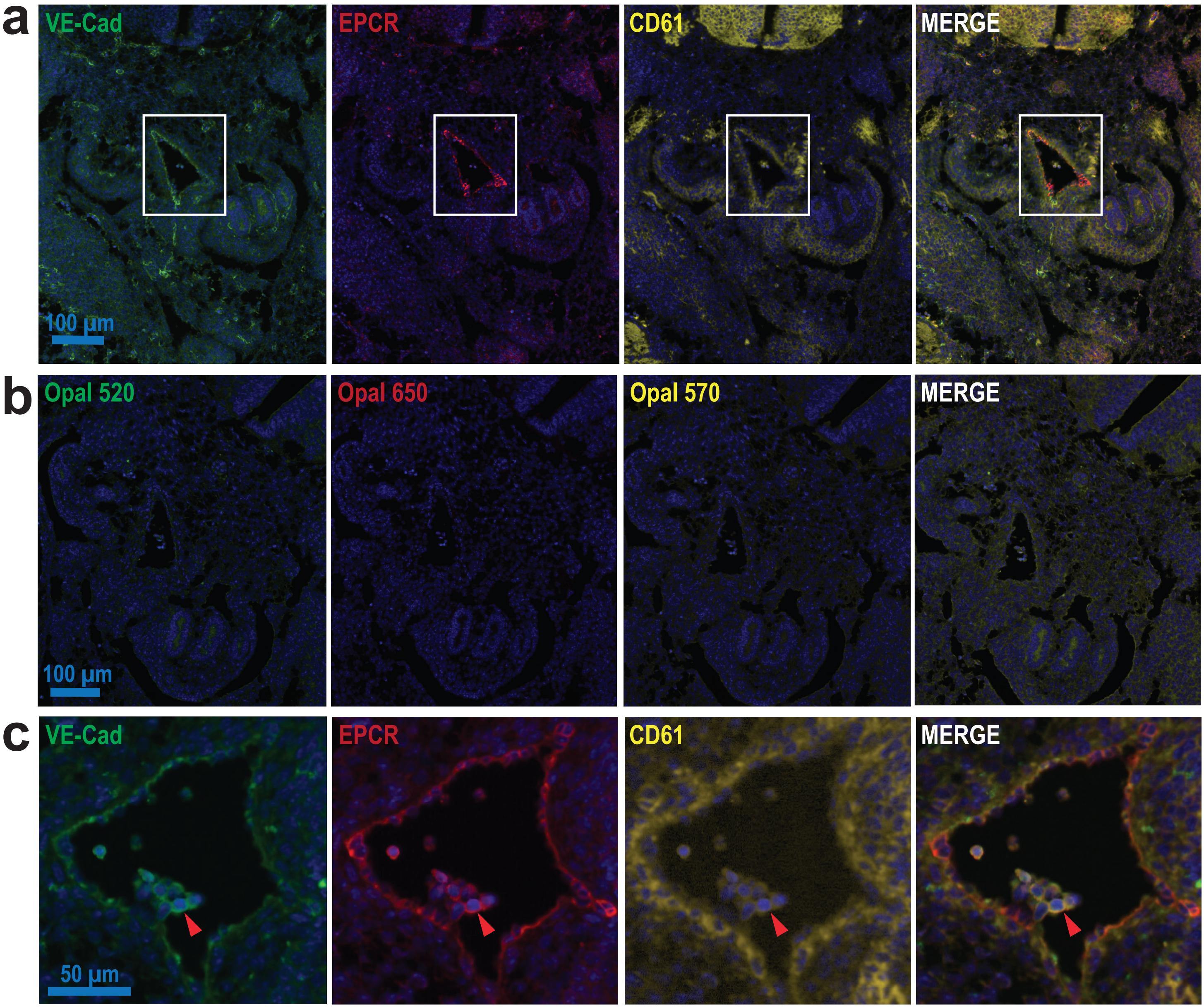

Supplementary Figure 3. a. Multiplexed immunostaining for VE-Cadherin, EPCR, and CD61 in the AGM region of E10.5 embryos (38-39 somite pairs). The aorta is indicated in the white box. All images counterstained with DAPI to show nuclei. **b.** Control staining (secondary and corresponding Opal Dye as indicated in absence of primary antibody) of section adjacent to section shown in (a). **c.** Aorta of the AGM region showing co-expression of VE-Cadherin, CD61, and EPCR heterogeneously within the intra-aortic hematopoietic clusters (red arrowhead). Similar results were obtained in sections from two independent experiments (embryos).

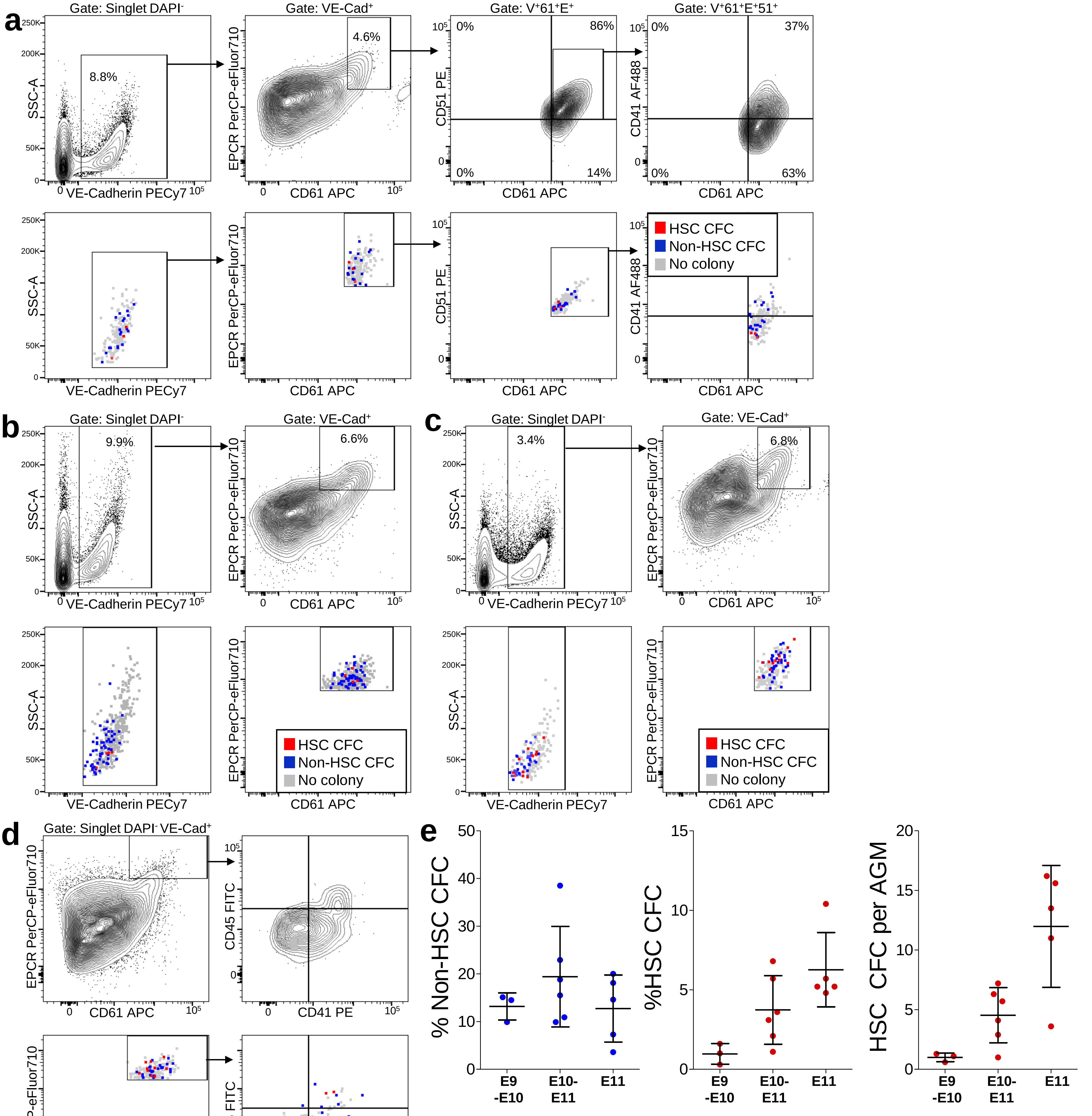

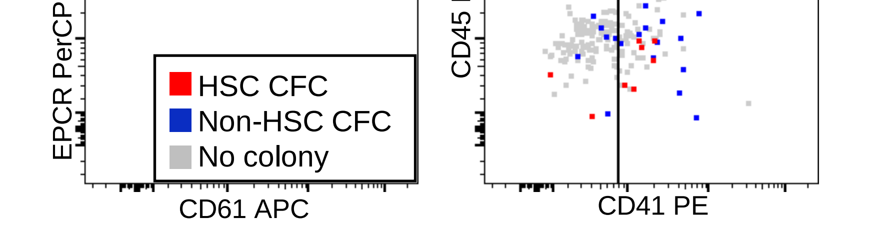

Stage	Somites	# Exp	%Non-HSC CFC	%HSC CFC	Est HSC CFC/AGM
E9-E10	26-32	3	13±2.8 (9.9-15)	1.0±0.7 (0.3-1.6)	1.0±0.4 (0.6-1.3)
E10-E11	30-40	6	19±11 (10-39)	3.7±2.2 (1.1-6.8)	4.5±2.3 (1.0-6.3)
E11	40-46	5	13±7.2 (3.6-20)	6.3±2.3 (4.8-10)	12±5.1 (3.6-16)

Supplementary Figure 4. a. Correlation of HSC CFC potential of individual index sorted V+61+E+ cells with surface expression of CD41 and CD51 at late E9 (26-30 sp). **b-c.** Representative experiments showing HSC CFC potential of individual index sorted V+61+E+ cells at (b) early E10 (30-32 sp) and (c) early E11 (40-42 sp). **d.** Correlation of HSC CFC potential of individual index sorted V+61+E+ cells with surface expression of CD41 and CD45 at late E10/early E11 (39-41 sp). **e.** Frequency of non-HSC CFC and HSC CFC within the V+61+E+ population and total HSC CFC detected per AGM across multiple, independent experiments at various developmental stages as indicated. Numbers in table indicate mean ± standard deviation, with range in parenthesis. (Related to Fig. 2c-d).

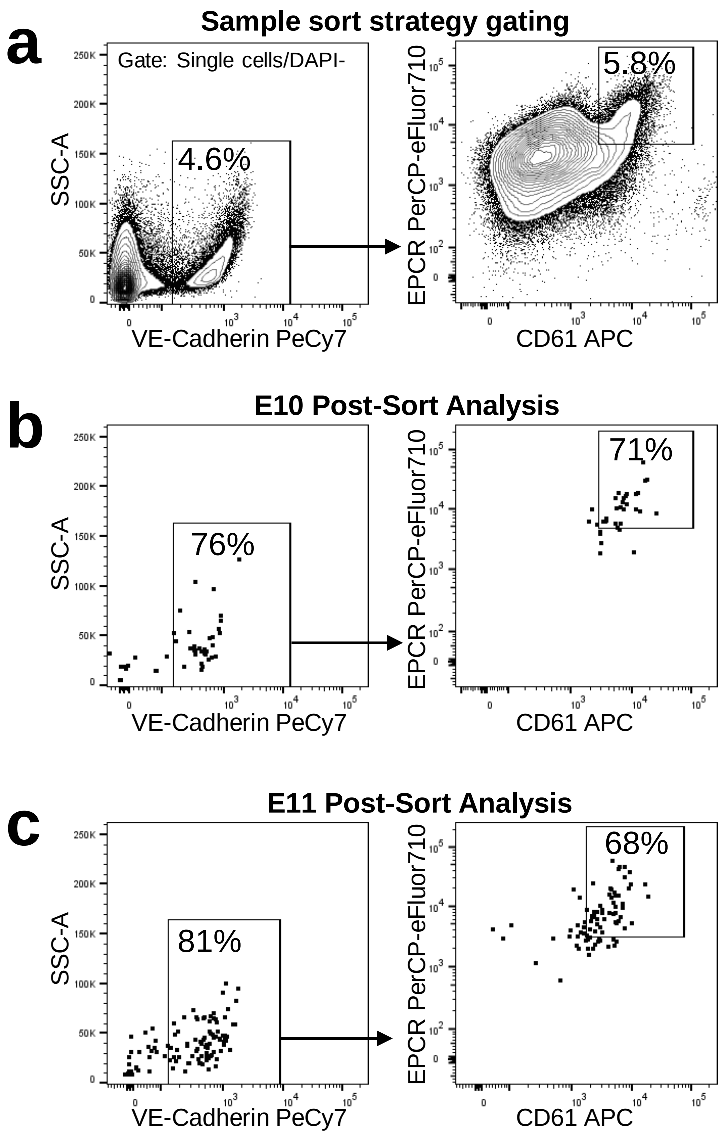

d

Sample	E10 (31-39 sp)	E11 (40-45 sp)
Embryos pooled	46	54
Cells sequenced	1622	1470

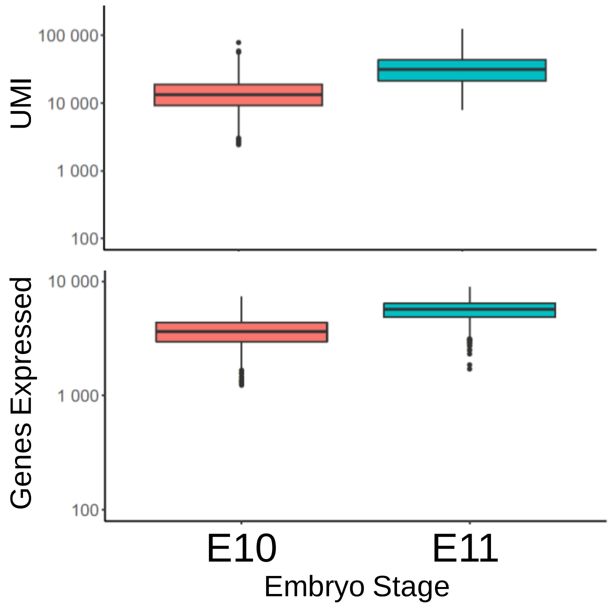

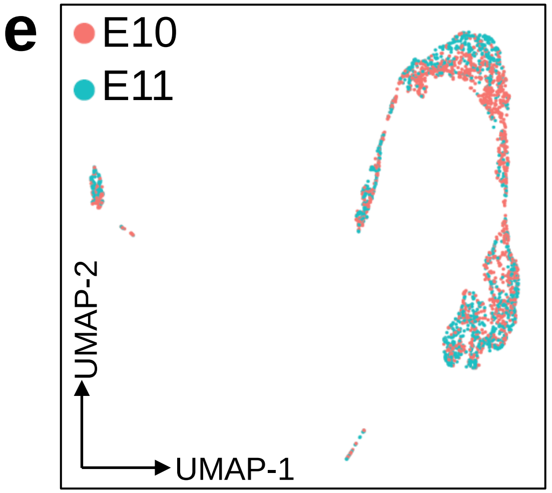

f

From Figure 3c

Cell type	Genes expressed	Genes not expressed
Arterial EC	*Cdh5, Dll4, Efnb2, & Hey1*	*Runx1, Gfi1, Nr2f2, Nrp2*
Other EC (non-arterial)	*Cdh5, Nr2f2, & Nrp2*	*Runx1, Gfi1, Efnb2, Hey1, Meox1*
Hematopoietic	*Runx1 & Gfi1*	
Somite	*Pax1 & Meox1* or *Pax3 & Myf5*	

From Figure 3h-j

Cell type	Genes expressed	Genes not expressed
HSC Precursor	*Gfi1, Mycn, Pdzk1ip1, Procr, Dll4, Vwf, Cdkn1c, Pbx1, & Mllt3*	*Flt3, Il7r, Fcgr3, Csf3r*
HE Subtype		Also not expressing *Itga2b, Spn, Ptprc*
Progenitor	*Runx1& Flt3, Il7r, Fcgr3,* or *Csf3r*	

g

Cluster 6 cells (see Fig 3a)

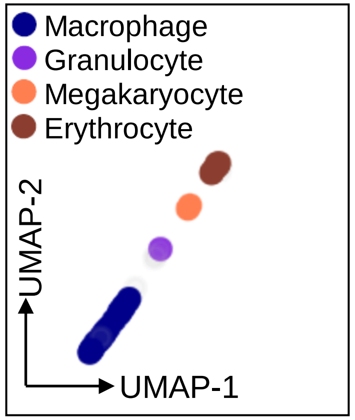

Cell type	Genes expressed
Macrophage	*C1qb, Mrc1*
Granulocyte	*Fcnb*
Megakaryocyte	*Pf4, Gata1*
Erythrocyte	*Gypa, Klf1*

Supplementary Figure 5. a-c. Sort strategy (a) and post-sort analysis for E10 (b) and E11 (c) AGM V+61+E+ cells isolated for scRNA-seq. **d.** Counts of UMI and unique genes expressed per cell for each embryo stage/sample. Boxplots show median values and interquartile ranges; upper/lower whiskers show 1.5X interquartile range with outliers shown as individual dots. **e**. UMAP with cells shown by embryo stage/sample. **f.** Genes used for cell type classification. **g.** Mature hematopoietic cell types identified in cluster 6. (Related to Fig. 3).

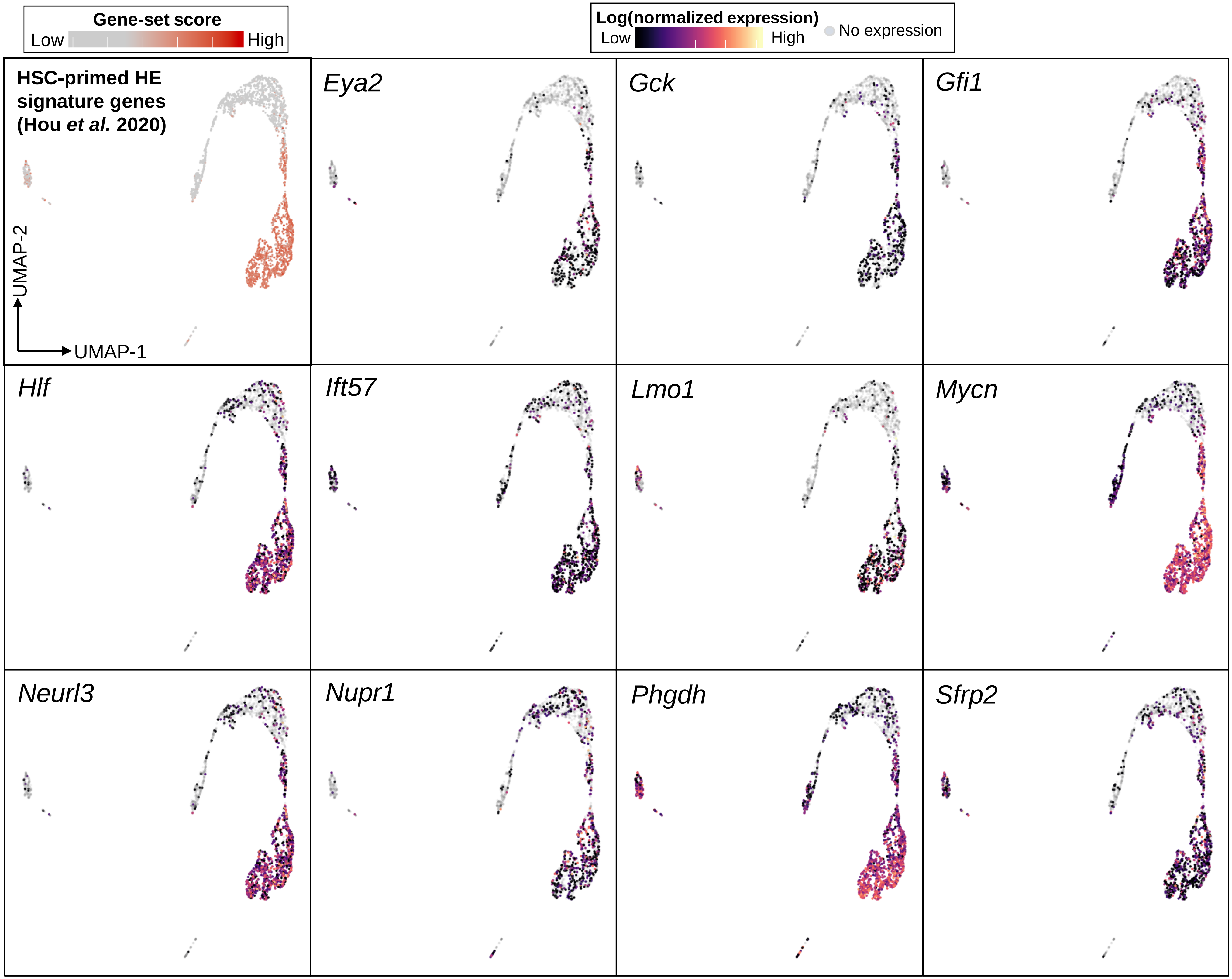

Supplementary Figure 6. Expression of genes contained within the HSC-primed HE signature gene set defined by Hou *et al.* (2020). (Related to Fig. 3f).

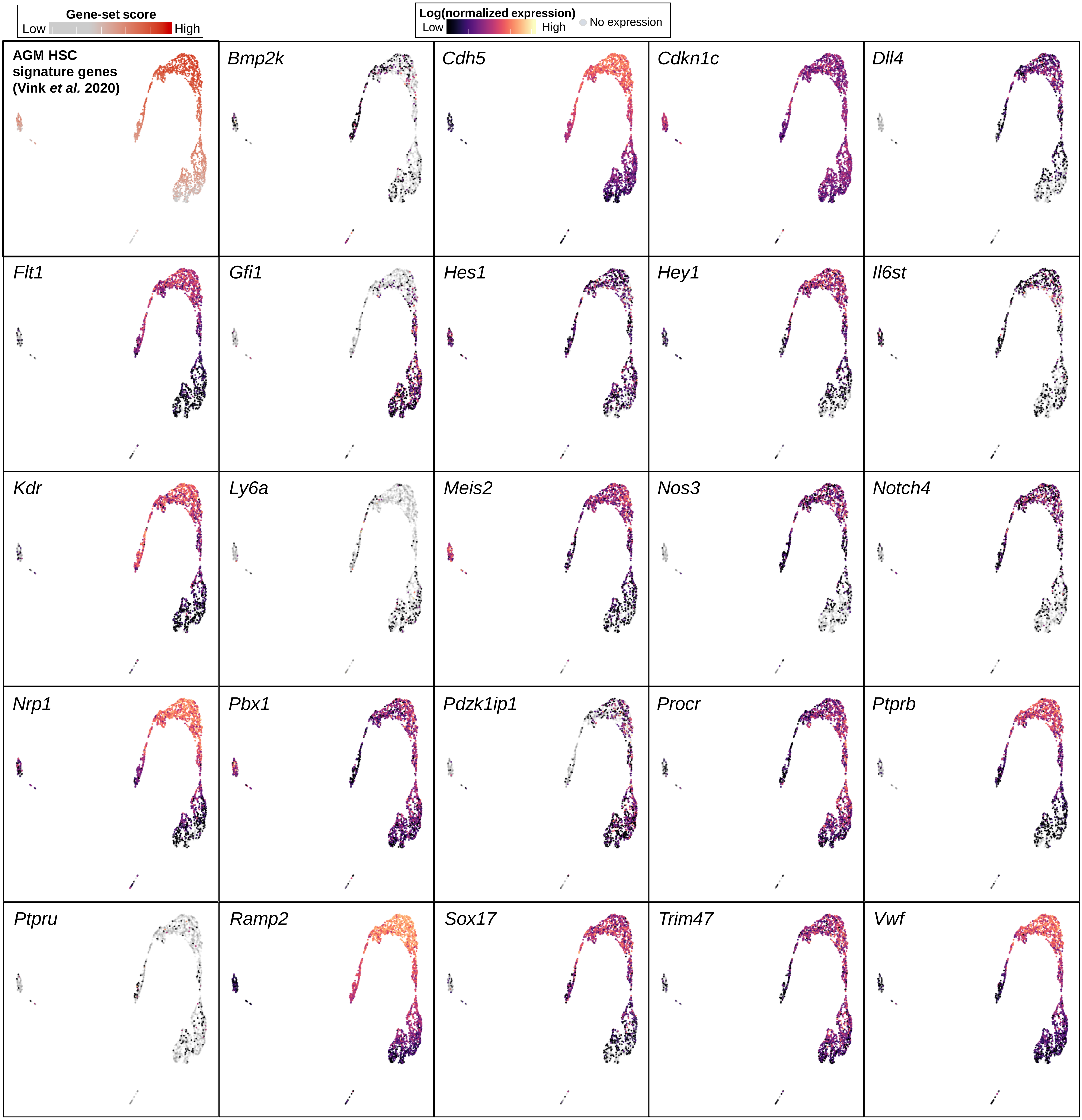

Supplementary Figure 7. Expression of genes contained within the E11 AGM HSC signature gene set defined by Vink *et al.* (2020). (Related to Fig. 3f).

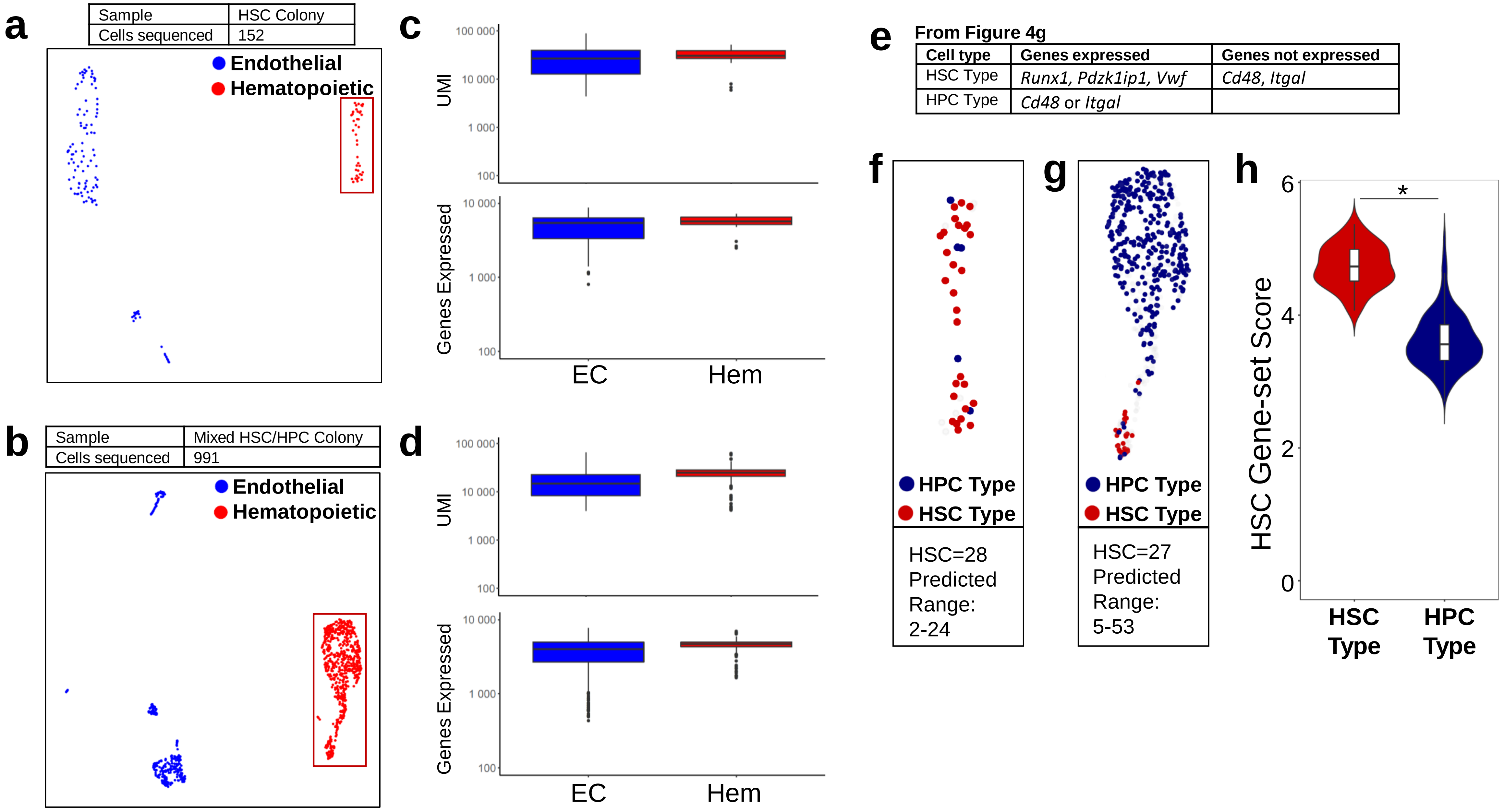

Supplementary Figure 8. a-b. UMAP of single cell transcriptomes of the progeny of a single V+61+E+ hemogenic precursor following co-culture on AGM-EC, with (a) "HSC" phenotype and (b) mixed "HSC/HPC" phenotype, showing clusters classified by cell type as endothelial (EC) and hematopoietic (Hem), based on expression of VE-Cadherin (*Cdh5)* and CD45 (*Ptprc*) (Related to Fig. 4a-d). **c-d.** Counts of UMI per cell and unique genes expressed per cell for each cell type for samples shown in a-b, respectively. Boxplots show median values and interquartile ranges, upper/lower whiskers show 1.5X interquartile range, outliers shown as individual dots. **e.** Genes used for cell type classification of HSC and HPC. **f-g.** Cell type classification of hematopoietic cells from (a-b) as HSC and HPC based on gene expression as described in (e). (Lower panel indicates total numbers of transcriptionally defined HSC and range of functional HSC predicted based on calculations using previously published limit dilution transplantation analysis of comparable populations, Hadland *et al.* 2017). **h.** Gene-set scores for HSC signature genes (from Wilson *et al.* 2015) in transcriptionally defined HSC and HPC from (f-g) (boxplots show median values and interquartile ranges; upper/lower whiskers show 1.5X interquartile range, *p < 1E-15, unpaired, two-sided Wilcoxon test). (Related to Fig. 4).

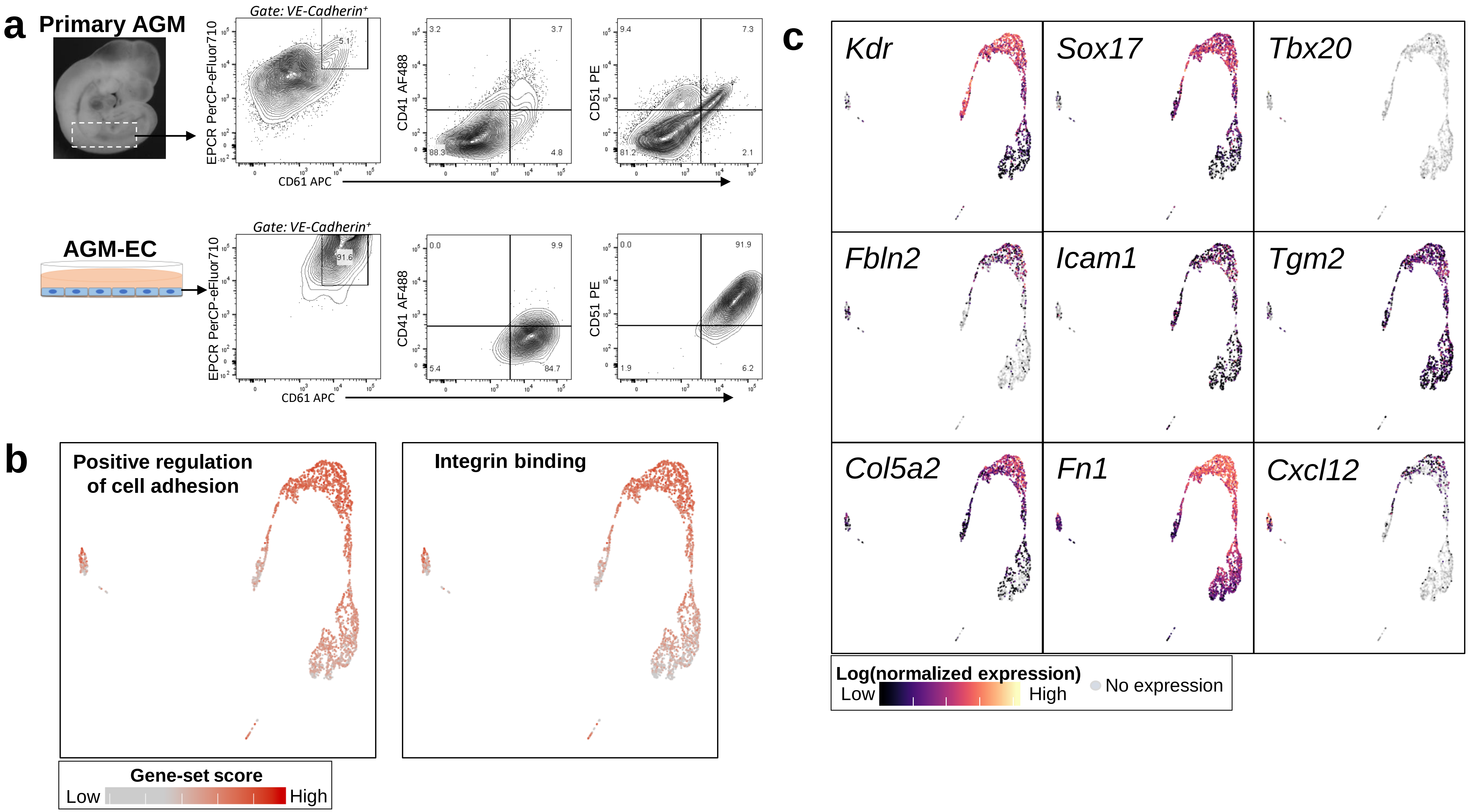

Supplementary Figure 9. Single cell RNA-sequencing of AGM V+61+E+ cells identifies transcriptional signatures of primary AGM niche arterial endothelial cells. a. Comparison of surface expression of EPCR, CD61, CD41, and CD51 in VE-Cadherin^{+} cells from primary E10 AGM and cultured AGM-EC. **b.** Gene set scores representing top GO terms for transcripts differentially expressed by HSC-supportive verses non-supportive AGM-EC in single cell transcriptomes from primary AGM V+61+E+ cells (see Fig. 1d). **c.** Gene expression heatmap in primary AGM V+61+E+ cells for endothelial marker, *Kdr,* and transcripts differentially expressed between HSC-supportive and non-supportive AGM-EC (see Fig. 1e). (Related to Fig. 1, Fig. 3).

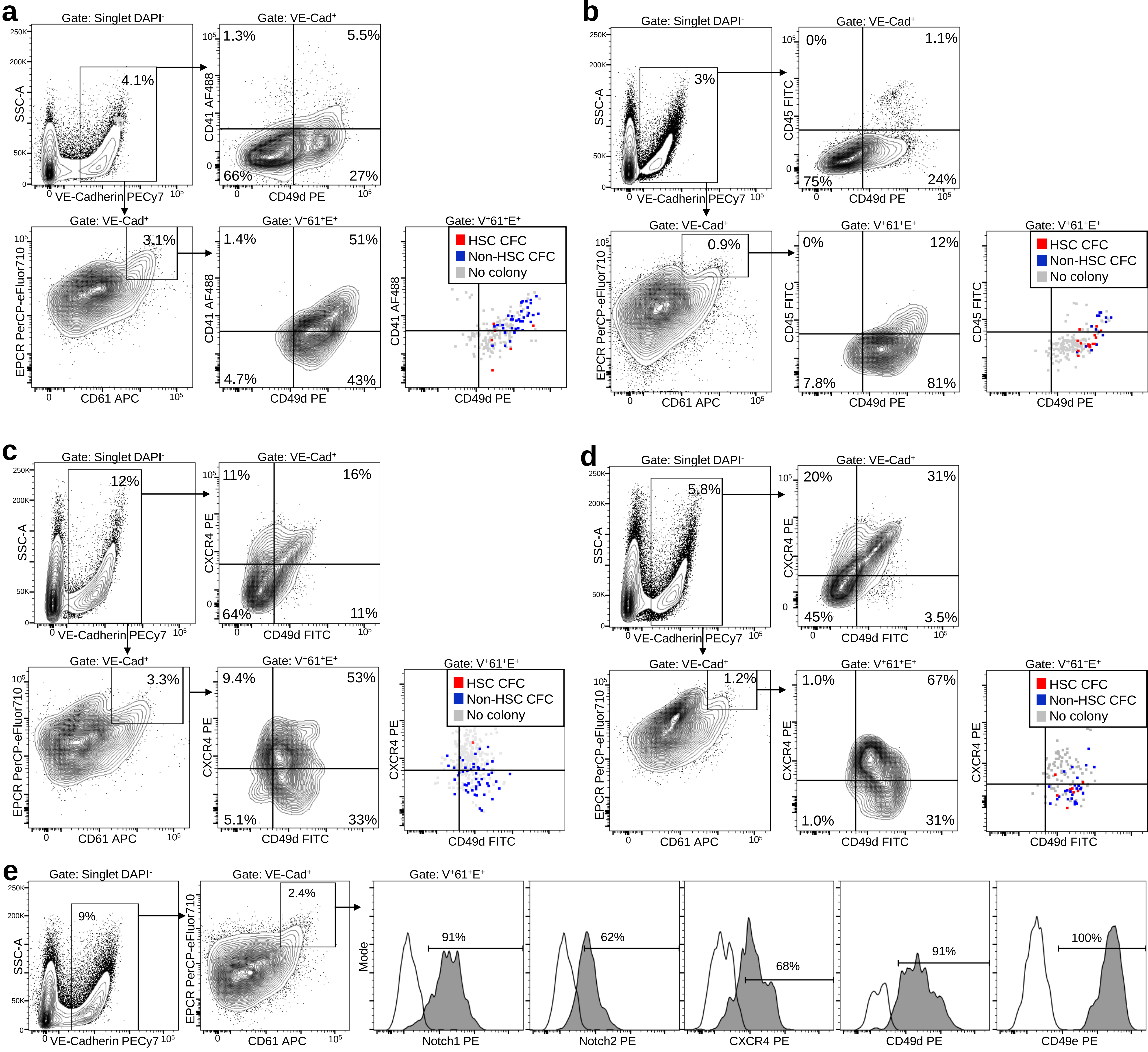

Supplementary Figure 10. Expression of receptors in AGM-derived HSC precursors by flow cytometry. **a-d.** Expression by flow cytometry in the total AGM-derived VE-cadherin^{+} population (upper panel) and the V+61+E+ subset (lower panel), and correlation with HSC CFC potential of index sorted cells (right lower panel) of: a) CD41 and CD49d at E10 (35-38 sp), b) CD45 and CD49d at late E10/early E11 (37-41 sp), c) CXCR4 and CD49d at E9 (~28 sp), and d) CXCR4 and CD49d at late E10 (38-39 sp).
e. Expression of cell surface receptors by flow in AGM-derived V+61+E+ cells at E10-E11 (30-41 sp). Unshaded histogram in each panel represents staining with relevant isotype controls. (Related to Fig. 5-6).

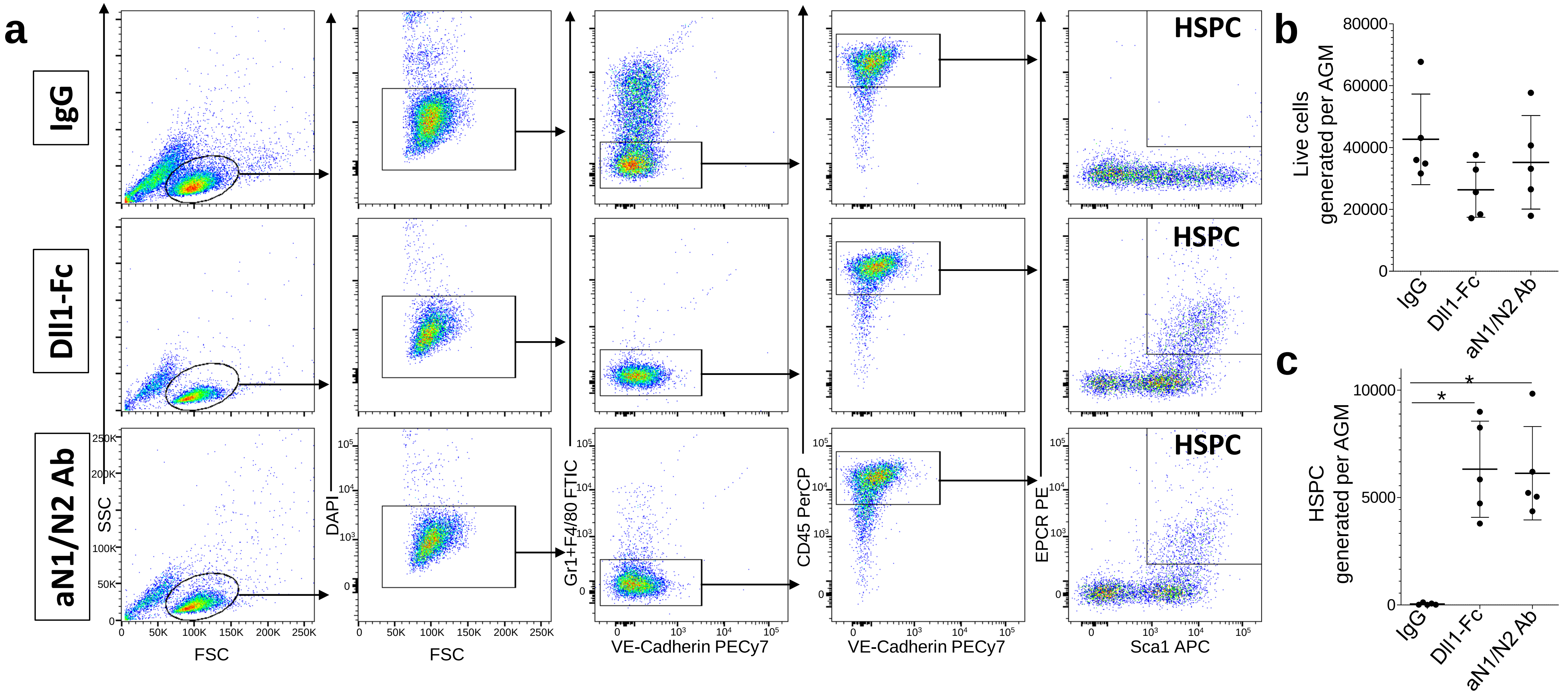

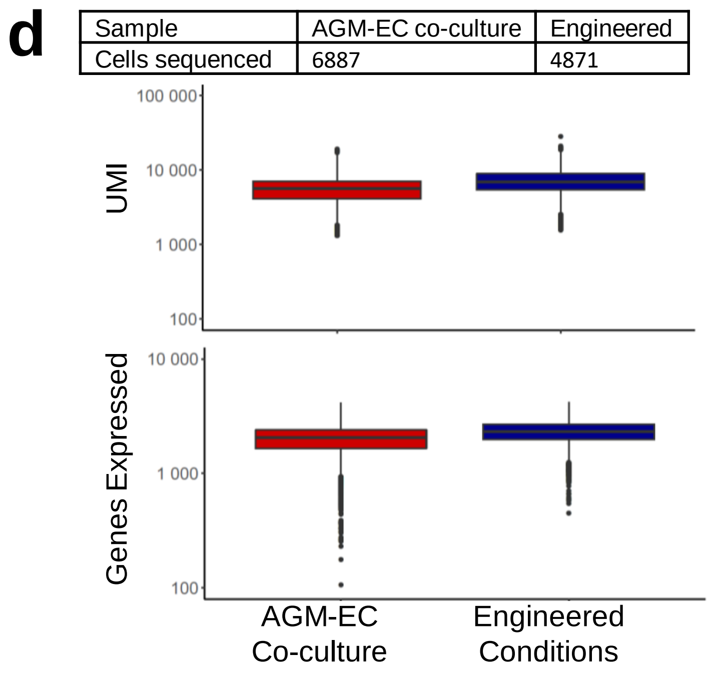

e From Figure 6f, 6h

Cell type	Genes expressed	Genes not expressed
HSC	*Pdzk1ip1, Hlf, Procr*	*Kdr*
MPP	*Kit, Cd34, Flt3*	*Pdzk1ip1*
T Prog	*Ptcra, Il7r*	
B Prog	*Ebf1, Il7r*	*Hlf, Pdzk1ip1*
Gran/Mono Prog	*Cd34, Fcgr3, & Cebpa, or Irf8 & Zeb2*	*Hlf, C1qb, Fcnb, Cd14, Gfi1*
Granulocyte	*Fcnb, Elane*	
Mono/Mac	*Cd14 & Adgre1, or C1qb & Cd36*	
Dendritic	*Siglech, Cd7*	
Mast	*Prss34, Cd200r3*	
Ery/Meg Prog	*Gata1, Gfi1b*	*Gypa, Cd34, Vwf, Prss34, Cd200r3*
Erythrocyte	*Gypa, Klf1*	
Megakaryocyte	*Vwf, Mpl*	*Hlf*
EC	*Cdh5, Kdr*	*Ptprc*

Supplementary Figure 11. a. Flow cytometric analysis of progeny of E11 AGM-derived V+61+E+ cells follow culture in engineered conditions in the presence of immobilized Dll1-Fc, aN1/N2 Ab, or control (IgG), showing gating for phenotypic population of hematopoietic stem and progenitor cells (HSPC) (VE-Cad$^{low/-}$CD45^{+}Gr1-F4/80^{-}Sca1^{+}EPCR^{+}). **b.** Total live cells and **c.** total HSPC generated per AGM equivalent of starting population following culture in engineered conditions in the presence of immobilized Dll1-Fc, aN1/N2 Ab, or control (IgG). (n=5 replicates per group, similar results obtained in n=3 independent experiments; error bars show median +/- SD. *p =0.0002, unpaired, two-sided Student's t-test) (Relate to Fig. 6b) (Source data are provided as a Source Data file). **d.** Counts of UMI per cell and unique genes expressed per cell for each sample. Boxplots show median values and interquartile ranges, upper/lower whiskers show 1.5X interquartile range, outliers shown as individual dots. **e.** Genes for cell type classification. (Related to Fig. 6e-h).

Supplementary Table 1. List of antibodies and key reagents.

**Antibody/Reagent**	**Vendor**	**Catalog #/reference**
CD201 (EPCR) Rat Anti-mouse Monoclonal Antibody PerCP-eFluor710 Clone eBio 1560	eBioscience	Cat#46-2012-80; RRID: AB_10718383
Rat IgG2b kappa Isotype Control Antibody PerCP-eFluor710 Clone eB149/10H5	eBioscience	Cat#46-4031-82; RRID: AB_1834457
CD61 Armenian Hamster Anti-mouse/rat Monoclonal Antibody APC Clone 2C9.G2	Biolegend	Cat#104316; RRID: AB_2561734
Armenian Hamster IgG Isotype Control Antibody APC Clone HTK888	Biolegend	Cat#400911
CD144 (VE-cadherin) Rat Anti-mouse Monoclonal Antibody PE-Cyanine7 Clone eBioBV13	eBioscience	Cat#25-1441-82; RRID: AB_2573402
Rat IgG1 kappa Isotype Control Antibody PE-Cyanine7 Clone eBRG1	eBioscience	Cat#25-4301-82; RRID: AB_470198
CD45 Rat Anti-mouse Monoclonal Antibody FITC Clone 30-F11	eBioscience	Cat#11-0451-85; RRID: AB_465051
Rat IgG2b kappa Isotype Control Antibody FITC Clone A95-1	BD Pharmingen	Cat#553988; RRID: AB_479619
CD41 Rat Anti-mouse Monoclonal Antibody Alexa Fluor 488 or PE Clone MWReg30	Biolegend	Cat#133908; RRID: AB_10645332
Rat IgG1 kappa Isotype Control Antibody Alexa Fluor 488 Clone RTK2071	Biolegend	Cat#400417; RRID: AB_389319
CD45 Rat Anti-mouse Monoclonal Antibody PE Clone 30-F11	eBioscience	Cat#12-0451-82; RRID: AB_465668
Gr-1 Rat Anti-mouse Monoclonal Antibody FITC Clone RB6-8C5	BD Pharmingen	Cat#553127; RRID: AB_394643
F4/80 Rat Anti-mouse Monoclonal Antibody FITC Clone BM8	Biolegend	Cat#123108; RRID: AB_893502
CD201 (EPCR) Rat Anti-mouse Monoclonal Antibody PE Clone eBio1560	eBioscience	Cat#12-2012-82; RRID: AB_914317
CD45 Rat Anti-mouse Monoclonal Antibody PerCP-Cyanine5.5 Clone 30-F11	eBioscience	Cat#45-0451-80; RRID: AB_906233
Ly-6A/E (Sca-1) Rat Anti-mouse Monoclonal Antibody APC Clone D7	eBioscience	Cat#17-5981-82; RRID: AB_469487
CD3 Rat Anti-Mouse Monoclonal Antibody FITC Clone 17A2	BD Pharmingen	Cat#555274; RRID: AB_395698
Rat IgG2b kappa Isotype Control Antibody PE Clone RTK4530	Biolegend	Cat# 400607;
F4/80 Rat Anti-mouse Monoclonal Antibody PE Clone BM8	eBioscience	Cat#12-4801-82; RRID: AB_465923
Rat IgG2a kappa Isotype Control Antibody PE Clone eBR2a	eBioscience	Cat#12-4321-83; RRID: AB_470053
Gr-1 Rat Anti-Mouse PerCP-Cy5.5 Clone RB6-8C5	BD Pharmingen	Cat#552093; RRID: AB_394334
Rat IgG2a kappa Isotype Control Antibody PerCP-Cy5.5 Clone RTK2758	Biolegend	Cat#400532
CD45.1 Mouse Anti-mouse Monoclonal Antibody PE-Cyanine7 Clone A20	eBioscience	Cat#25-0453-82; RRID: AB_469629
CD19 Rat Anti-mouse Monoclonal Antibody APC Clone 1D3	BD Pharmingen	Cat#550992; RRID: AB_398483
Rat IgG2a kappa Isotype Control Antibody APC Clone R35-95	BD Pharmingen	Cat#553932; RRID: AB_479720
CD45.2 Mouse Anti-mouse Monoclonal Antibody APC-eFluor 780 Clone 104	eBioscience	Cat#47-0454-82; RRID: AB_1272175

Rat IgG2a kappa Isotype Control Antibody APC-eFluor 780 Clone eBR2a	eBioscience	Cat#47-4321-82; RRID: AB_1271997
CD43 Rat Anti-mouse Monoclonal Antibody FITC Clone eBioR2/60	eBioscience	Cat#11-0431-81 RRID: AB_465039
Rat IgM Isotype Control Antibody FITC	eBioscience	Cat#11-4341-82 RRID: AB_470017
CD49d Rat Anti-mouse Monoclonal Antibody PE Clone R1-2	Biolegend	Cat#103607; RRID: AB_313038
CD49d Rat Anti-mouse Monoclonal Antibody FITC Clone R1-2	Biolegend	Cat#103605; RRID: AB_313036
CD184 (CXCR4) Rat Anti-mouse Monoclonal Antibody PE Clone 2B11	eBioscience	Cat#12-9991-81; RRID: AB_891393
CD44 Rat Anti-mouse Monoclonal Antibody FITC Clone IM7	BD Pharmingen	Cat#561859
CD117/c-kit Rat Anti-mouse Monoclonal Antibody FITC Clone 2B8	BD Pharmingen	Cat#561680 RRID: AB_394805
AA4.1 Rat Anti-mouse Monoclonal Antibody FITC Clone AA4.1	BD Pharmingen	Cat#561990
ESAM Rat Anti-mouse Monoclonal Antibody FITC Clone IG8/ESAM	Biolegend	Cat#136205
Notch 1 Armenian Hamster Anti-mouse Monoclonal Antibody PE Clone HMN1-12	Biolegend	Cat#130607
Notch 2 Armenian Hamster Anti-mouse Monoclonal Antibody PE Clone HMN2-35	Biolegend	Cat#130707
Armenian Hamster IgG Isotype Control Antibody PE Clone HTK888	Biolegend	Cat#400907
CD49e Rat Anti-mouse Monoclonal Antibody PE Clone 5H10-27 (MFR5)	BD Pharmingen	Cat#557447
CD105/Endoglin Rat Anti-mouse Monoclonal Antibody PE clone MJ7/18	Biolegend	Cat#120407
CD51 Rat Anti-mouse Monoclonal Antibody PE Clone RMV-7	BD Pharmingen	Cat#551187
CD133 Rat Anti-mouse Monoclonal Antibody PE Clone 13A4	eBioscience	Cat#14-1331-82 RRID: AB_467471
DLL4 Armenian Hamster Anti-mouse Monoclonal Antibody PE Clone HMD4-1	Biolegend	Cat#130807
VE-Cadherin Goat Anti-mouse Polyclonal Antibody Unconjugated	R&D Systems	Cat#AF1002-SP
EPCR Rabbit Anti-mouse Polyclonal Antibody Unconjugated	Invitrogen	Cat#PA5-81459
CD61 Rabbit Anti-mouse Monoclonal Antibody Unconjugated Clone SJ19-09	Novus	Cat#NBP2-67416
Notch 1 Antibody Ultra-LEAF Purified anti-mouse Clone HMN1-12	Biolegend	Part No 95499
Notch 2 Antibody Ultra-LEAF Purified anti-mouse Clone HMN2-35	Biolegend	Part No 96014
Armenian Hamster IgG Isotype Ctrl Antibody Ultra-LEAF Purified Clone HTK-888	Biolegend	Part No 400940
Human Delta-like-1 Fc chimera (Dll1-Fc)	In house	
Human IgG Fc control	In house	
RetroNectin (r-Fibronectin CH-296)	Takara	Cat#T100B
Recombinant Mouse VCAM-1/CD106 Fc Chimera	R&D Systems	Cat#643-VM-200
Recombinant Murine SCF	Peprotech	Cat#250-03
Recombinant Murine IL-3	Peprotech	Cat#213-13

Recombinant Human TPO	Peprotech	Cat#300-18
Recombinant Mouse CXCL12/SDF-1a	R&D Systems	Cat#460-SD-050
LY 364947	Tocris	Cat#2718
SB431542	Tocris	Cat#1614
CHIR99021	Stemgent	Cat#04-0004-10
Endothelial mitogen (ECGS)	Alfa Aesar	Cat#BT-203
Heparin sodium salt	Sigma	Cat#H3149-100KU
TrypLE Express	Gibco	Cat#12605-028
Collagenase Type 1 (0.25%)	Stemcell Technologies	Cat#07902
Ultrapure Bovine Serum Albumin	ThermoFisher Scientific	
Fetal Bovine Serum	ThermoFisher	Special order characterized lot
X-VIVO 20	Lonza	Cat#04-448Q
StemSpan SFEM II	Stemcell Technologies	Cat#09655
EmbryoMax 0.1% Gelatin solution	MilliporeSigma	Cat#ES006B
Neutral-buffered formalin solution (10%)	Sigma-Aldrich	Cat#HT5011
