# Supplementary material for: Engineering a niche supporting hematopoietic stem cell development using integrated single-cell transcriptomics
Source: Nat Commun. 2022 Mar 24;13:1584. doi: 10.1038/s41467-022-28781-z (PMC8948249; doi:10.1038/s41467-022-28781-z)
Supplement: Supplementary file 2 — Description of Additional Supplementary Files [file 41467_2022_28781_MOESM2_ESM.pdf]

Description of Additional Supplementary Data Files

Supplementary Data 1. Cluster-specific genes differentially expressed in HSC-supportive AGM-EC (cluster 1) or non-supportive AGM-EC (Cluster 2), ordered by specificity. (Related to Fig. 1c-e).

Supplementary Data 2. Gene ontology terms (biological processes and molecular functions) associated with cluster-specific genes expressed in HSC-supportive AGM-EC. List of cluster-specific genes used for aggregated gene scores based on gene ontology terms. (Related to Fig. 1d).

Supplementary Data 3. Genes differentially expressed over pseudotime representing the EC/HE to hematopoietic transition, ordered by q-value. (Related to Fig. 3d-e).

Supplementary Data 4. Genes differentially expressed over pseudotime representing HSC to HPC differentiation, ordered by q-value. (Related to Fig. 4h).

Supplementary Data 5. List of ligand-receptor interactions between primary AGM-derived arterial EC or HSC-supportive AGM-EC stroma and primary AGM-derived HE/pre-HSC or in vitro generated HSC (Related to Fig. 5).

Supplementary Data 6. Signature/marker genes from published studies of HE, pre-HSC, AGM HSC, and adult HSC used for gene-set scores (Related to Fig. 3).
