# Supplementary material for: Engineering a niche supporting hematopoietic stem cell development using integrated single-cell transcriptomics
Source: Nat Commun. 2022 Mar 24;13:1584. doi: 10.1038/s41467-022-28781-z (PMC8948249; doi:10.1038/s41467-022-28781-z)
Supplement: Supplementary file 9 — Reporting Summary [file 41467_2022_28781_MOESM9_ESM.pdf]

nature research

Corresponding author(s): Brandon Hadland

Last updated by author(s): December 20, 2021

Reporting Summary

Nature Research wishes to improve the reproducibility of the work that we publish. This form provides structure for consistency and transparency in reporting. For further information on Nature Research policies, see our Editorial Policies and the Editorial Policy Checklist.

Statistics

For all statistical analyses, confirm that the following items are present in the figure legend, table legend, main text, or Methods section.

n/a	Confirmed	
☐	☒	The exact sample size (*n*) for each experimental group/condition, given as a discrete number and unit of measurement
☐	☒	A statement on whether measurements were taken from distinct samples or whether the same sample was measured repeatedly
☐	☒	The statistical test(s) used AND whether they are one- or two-sided<br>*Only common tests should be described solely by name; describe more complex techniques in the Methods section.*
☒	☐	A description of all covariates tested
☒	☐	A description of any assumptions or corrections, such as tests of normality and adjustment for multiple comparisons
☐	☒	A full description of the statistical parameters including central tendency (e.g. means) or other basic estimates (e.g. regression coefficient) AND variation (e.g. standard deviation) or associated estimates of uncertainty (e.g. confidence intervals)
☐	☒	For null hypothesis testing, the test statistic (e.g. *F*, *t*, *r*) with confidence intervals, effect sizes, degrees of freedom and *P* value noted<br>*Give P values as exact values whenever suitable.*
☒	☐	For Bayesian analysis, information on the choice of priors and Markov chain Monte Carlo settings
☒	☐	For hierarchical and complex designs, identification of the appropriate level for tests and full reporting of outcomes
☒	☐	Estimates of effect sizes (e.g. Cohen's *d*, Pearson's *r*), indicating how they were calculated

Our web collection on statistics for biologists contains articles on many of the points above.

Software and code

Policy information about availability of computer code

Data collection	No software was used to collect the data.
Data analysis	Cell Ranger 2.1.1 pipeline (10X Genomics), PANTHER Overrepresentation Test (Released 2019-07-11, GO Ontology database Released 2019-07-03), R v3.6.1 (R packages: Monocle v.2.99.3, ggplot2 v 3.3.2, mygene v1.20.0, ggpubr v0.4.0, circlize v0.4.8, iTalk v0.1.0), FloJo (version 10.1), FACSDiva v9

For manuscripts utilizing custom algorithms or software that are central to the research but not yet described in published literature, software must be made available to editors and reviewers. We strongly encourage code deposition in a community repository (e.g. GitHub). See the Nature Research guidelines for submitting code & software for further information.

Data

Policy information about availability of data

All manuscripts must include a data availability statement. This statement should provide the following information, where applicable:

- Accession codes, unique identifiers, or web links for publicly available datasets
- A list of figures that have associated raw data
- A description of any restrictions on data availability

All sequencing data is publicly available at NCBI GEO (Accession number GSE145886).

Field-specific reporting

Please select the one below that is the best fit for your research. If you are not sure, read the appropriate sections before making your selection.

☒ Life sciences ☐ Behavioural & social sciences ☐ Ecological, evolutionary & environmental sciences

For a reference copy of the document with all sections, see nature.com/documents/nr-reporting-summary-flat.pdf

Life sciences study design

All studies must disclose on these points even when the disclosure is negative.

Sample size	For transplant experiments comparing conditions, a minimum of 5 mice were transplanted per condition, in a minimum of 2 independent experiments (as described in the text, with pooled results from independent experiments shown for most experiments, as indicated). This sample size was chosen as it is sufficient to measure significant differences in engraftment in experiments similar to those in this study (ie. where minimal or no engraftment is observed in the control conditions) and to ensure reproducibility. For single cell transcriptomic experiments, the number of cells captured for each analysis (as indicated in methods and supplementary figures 1, 5, 8, 11) was determined by the expected frequency of populations or cell types of interest (egs. clonal HSC precursors in AGM samples or HSC in colonies following AGM-EC culture), in order to capture sufficient numbers of rare cells for robust analysis. For all experiments, multiple embryos were pooled (as indicated in the text) from litters at equivalent stages based on counting somite pairs, as indicated.
Data exclusions	Exclusion of poor quality/low UMI single cell transcriptomic data was performed independent of sample identity using default criteria in the 10X Genomics CellRanger pipeline (pertains to scRNAseq data in Fig 1c-e, Fig 3, Fig 4, Fig 5e-h, Sup Fig 1, 5, 6, 7, 8, 9, 11d-e) . Following dimensionality reduction and clustering in Monocle, clusters representing contaminating cell populations based on cell type classification were excluded for downstream analysis, as indicated in the text (pertains to data in Fig 3, Fig 4c-d, Sup Fig 5, 8).
Replication	Replicate, independent experiments (2 or greater) were performed for all assays (flow cytometry, transplantation) at different developmental stages as indicated in the text. Representative or pooled results from independent experiments where replication was successful are shown, as indicated. For single cell index analysis of primary AGM cells, at least two independent experiments were performed for each analysis at different embryonic stages, as indicated. For scRNA-seq studies, multiple independent AGM-EC lines, primary AGM samples from pooled embryos at two independent time points (E10 and E11) and two representative colony types following AGM-EC culture were analyzed, as indicated.
Randomization	For all transplant experiments, mice were randomly distributed amongst experimental groups.
Blinding	No blinding was performed for samples, though outcomes were measured quantitatively using identical methods (egs. peripheral blood engraftment, flow cytometry, gene expression measurements) and relevant controls (egs. isotype staining) independent of sample identity.

Reporting for specific materials, systems and methods

We require information from authors about some types of materials, experimental systems and methods used in many studies. Here, indicate whether each material, system or method listed is relevant to your study. If you are not sure if a list item applies to your research, read the appropriate section before selecting a response.

Materials & experimental systems

n/a	Involved in the study	
☐	☒	Antibodies
☐	☒	Eukaryotic cell lines
☒	☐	Palaeontology and archaeology
☐	☒	Animals and other organisms
☒	☐	Human research participants
☒	☐	Clinical data
☒	☐	Dual use research of concern

Methods

n/a	Involved in the study	
☒	☐	ChIP-seq
☐	☒	Flow cytometry
☒	☐	MRI-based neuroimaging

Antibodies

Antibodies used	List of all antibodies, clones, and catalog numbers are provided in Supplementary Data 7.
Validation	For antibodies used for flow cytometry in this study, staining was compared to relevant isotype controls; and compensation accounted using single-positive controls and fluorescence-minus-one controls. Concentration of each antibody and staining conditions were per manufacturer's recommendations, as described in the methods. Species specificity for each antibody clone and validation for application to flow cytometry are described on the manufacturer's website.

Eukaryotic cell lines

Policy information about cell lines

Cell line source(s)	The methods for derivation of cultured endothelial cells (AGM-EC) in this study are described in the methods, and as

Cell line source(s)	indicated in the text in previous publications. A detailed protocol was also submitted to Nature Protocol Exchange (Generation of AGM-derived Akt-EC, Dignum et al).
Authentication	Authentication of AGM-EC is described in PMID: 25866967 (Hadland et al JCI, 2015) and in a protocol submitted to Nature Protocol Exchange (above). All endothelial lines are routinely testing by flow cytometry for relevant endothelial markers (VE-cadherin, Flk1, CD31) to ensure purity.
Mycoplasma contamination	AGM-EC used in this study have tested negative for mycoplasma contamination
Commonly misidentified lines (See ICLAC register)	No commonly misidentified lines were used in this study.

Animals and other organisms

Policy information about studies involving animals; ARRIVE guidelines recommended for reporting animal research

Laboratory animals	Wild type C57Bl6/J7 (CD45.2) and congenic C57BL/6.SJL-Ly5.1-Pep3b (CD45.1) strain mice were used for all studies at 6-10 weeks of age. For transplantation experiments, both male and females were used as recipients. For each experiment, sexes were distributed equivalently across conditions. For embryo studies, pooled embryos were used for all experiments independent of sex.
Wild animals	The study did not involve wild animals.
Field-collected samples	The study did not involve samples from the field.
Ethics oversight	All animal studies were conducted in accordance with the NIH guidelines for humane treatment of animals and were approved by the Institutional Animal Care and Use Committee at the Fred Hutchinson Cancer Research Center.

Note that full information on the approval of the study protocol must also be provided in the manuscript.

Flow Cytometry

Plots

Confirm that:

☒ The axis labels state the marker and fluorochrome used (e.g. CD4-FITC).

☒ The axis scales are clearly visible. Include numbers along axes only for bottom left plot of group (a 'group' is an analysis of identical markers).

☒ All plots are contour plots with outliers or pseudocolor plots.

☒ A numerical value for number of cells or percentage (with statistics) is provided.

Methodology

Sample preparation	Processing of AGM-derived cells and cultured cells for flow cytometry is described in the methods. Briefly, embryo samples were treated with collagenase and dissociated by pipetting. Cultured cells were harvested by TrypLE (AGM-EC) or by vigorous pipetting (co-cultured hematopoieitic cells). All antibody staining and flow was performed on ice in PBS containing FBS.
Instrument	BD FACSAria II, Canto 2
Software	BD FACSDiva Software, FlowJo
Cell population abundance	Details provided for various samples analyzed at each embryonic stage in the manuscript. Sample sorting strategy and post-sort purity for primary AGM samples at E10 and E11 are provided in Sup Figure 4.
Gating strategy	Gates for positive staining cell populations were determined based on relevant isotype controls; and compensation was adjusted using single-positive controls. For staining within a subpopulation, fluorescence-minus-one controls were used to set gates (for egs. see Sup Fig. 2a, 2d). All axes are labeled with relevant antibodies (fluorochromes provided in methods and Sup. Table 8).

☒ Tick this box to confirm that a figure exemplifying the gating strategy is provided in the Supplementary Information.
